# Sophocarpine Inhibits Proliferation and Triggers Apoptosis in Breast Cancer Cells by Modulating the miR-21/PTEN/AKT and ERK1/2 Signaling Networks

**DOI:** 10.3390/ph19101513

**Published:** 2026-09-23

**Authors:** Yasmin R. Shahin, Nihal M. Elguindy, Doaa A. Ghareeb, Seham Elabd, Mahmoud Balbaa

**Affiliations:** 1 Department of Biochemistry, Faculty of Science, Alexandria University, Alexandria 21511, Egypt; yasmin.shahin@alexu.edu.eg (Y.R.S.); nihal.elguindy@alexu.edu.eg (N.M.E.); d.ghareeb@alexu.edu.eg (D.A.G.); 2 Department of Physiology, Medical Research Institute, Alexandria University, Alexandria 21511, Egypt; sehamelabd@gmail.com

**Keywords:** sophocarpine, alkaloid scaffold, HIF1-alpha, lncRNAGAS5, PTEN, PDCD4

## Abstract

**Background**: Sophocarpine (SC), a tetracyclic quinolizidine alkaloid, has shown antitumor potential, yet its activity and underlying mechanisms in breast cancer remain unexplored. This study aimed to investigate the anticancer effects of SC and the molecular mechanism, focusing on the involvement of microRNA-21 (miR-21) and AKT/mTOR and ERK1/2 signaling networks. **Methods**: In MDA-MB-231 and T-47D breast cancer cells, SC cytotoxicity and growth-inhibitory effects were evaluated using MTT viability, colony formation, migration assays, and flow cytometry for cell cycle and apoptosis analysis. RT-qPCR analysis, Western blotting, and immunofluorescence staining were performed to measure RNA and protein expression, respectively. Molecular docking analysis was performed to explore SC’s interaction with the miR-21 precursor. **Results**: SC treatment effectively inhibited the proliferation, survival, and migration of breast cancer cells. It also induced cell cycle arrest and triggered apoptosis in both cell lines. Mechanistically, SC downregulated oncogenic miR-21 transcription, thereby enhancing tumor suppressors PTEN and PDCD4 and suppressing AKT/mTOR and ERK1/2 signaling. Interestingly, restoration of miR-21 reversed SC’s effects on PTEN and PDCD4, confirming SC’s miR-21 dependency. Docking analysis suggested that SC may bind the miR-21 precursor and interfere with its maturation. SC also increased long noncoding RNA GAS5 while reducing miR-196a-5p and HIF-1α transcripts. It shifted the Bcl2/Bax/Bad/caspases balance toward apoptosis. **Conclusions**: These findings identify SC as a potential structural scaffold exhibiting preliminary anti-tumor activity against breast cancer cells, at least in part, through modulating miR-21 and inhibition of the AKT/mTOR/ERK1/2 survival network.

## 1. Introduction

Breast cancer is a serious public health challenge on a global scale, representing the leading cause of cancer deaths among women, with an estimated 2.3 million new cases globally in 2022 [1,2]. Numerous genetic changes in mammary epithelial cells can cause breast cancer [3]. These alterations strongly affect cellular signaling pathways in these cases [4]. Moreover, the ERK1/2 pathway often interacts with PI3K/AKT/mTOR signaling to drive more aggressive phenotypes [5]. These signaling pathways are critically implicated in the development, proliferation, evasion of apoptosis, angiogenesis, and metastasis of breast cancer, and their hyperactivation is strongly associated with poor prognosis and resistance of cancer cells to antitumor therapies [6].

Noncoding RNAs control several signaling pathways in cancer cells, resulting in the formation of complex regulatory networks. MicroRNAs are small noncoding RNAs that are created when the Dicer enzyme cleaves precursor miRNA (pre-miRNA) after transcription of primary miRNA (Pri-miRNA) [7]. They function as negative gene regulators by inhibiting translation and degrading mRNAs to repress the expression of the target genes [8]. MiRNA-21 (miR-21), as the most overexpressed miRNA in the great majority of malignancies, targets many important tumor suppressors to encourage breast cancer growth, proliferation, migration, and metastasis [9].

miR-21 regulates AKT/mTOR and ERK1/2 signaling through direct targeting of tumor suppressors such as PTEN (phosphatase and tensin homolog) and PDCD4 (programmed cell death 4) [10]. PTEN, a dual lipid and protein phosphatase, dephosphorylates PIP3 and thereby antagonizes PI3K/AKT signaling [11]. Loss of PTEN expression occurs in up to 48% of breast cancers and correlates with poor prognosis [12]. Similarly, PDCD4 expression is frequently reduced in breast tumors; PDCD4 suppresses tumor progression by inhibiting translation of oncogenic proteins and is regulated by mTOR activity during mitogenic stimulation [13]. Conversely, mTOR and ERK1/2 pathways modulate miR-21 expression and activity via multiple mechanisms. For example, the long noncoding RNA Growth Arrest Specific 5 (lncRNA GAS5), acting downstream of AKT/mTOR signaling, functions as a tumor suppressor by sequestering miR-21 and thereby reducing its oncogenic effects in breast cancer cells [14]. In addition, hypoxia-inducible factor-1 alpha (HIF-1α), whose abundance and activity are partly controlled by mTOR and ERK signaling, promotes miR-21 expression in breast cancer cells. This provides an additional mechanistic link between these signaling pathways and miR-21–mediated oncogenic activity [15,16]. Consistently, inhibition of miR-21 in several preclinical models has been reported to suppress tumor growth, induce cancer cell death, and reduce resistance to anticancer therapies [17,18].

Chemotherapy is commonly used as a treatment strategy for patients with triple-negative breast cancer (TNBC) and for those whose tumors have developed resistance to endocrine therapy [19]. However, its clinical effectiveness is often limited by severe adverse effects and the progressive development of drug resistance in cancer cells. In contrast, numerous natural agents derived from diverse biological sources have demonstrated promising anticancer activity in experimental studies, with the potential to reduce toxicity and overcome or delay therapeutic resistance across different tumor models. These drugs work against cancer in a number of ways, such as by inhibiting the growth, invasion, and metastasis of tumor cells, altering epigenetic processes, and controlling the expression of microRNA [20].

Sophocarpine (SC) is a matrine-type alkaloid and one of the major bioactive constituents of *Sophora alopecuroides* L. It is also isolated from the roots of *Sophora flavescens* and the aerial parts of *Sophora tonkinensis* [21]. Previous studies have shown that SC possesses several pharmacological effects, including antiviral, anti-tumor, anti-inflammatory, anti-nociceptive, neuroprotective, and immune regulatory effects [21]. Additionally, direct cytotoxic actions against many cancer cell types have been observed for SC. For instance, SC enhanced apoptosis in castration-resistant prostate cancer cells and reduced tumor tissue volume by inactivating the PI3K/AKT/mTOR pathway [22]. In colorectal cancer, SC markedly decreased the levels of inflammatory mediators and suppressed the MAPK signaling pathway [23]. In addition, SC derivatives, such as SC trifluorohydrazone, have been shown to inhibit proliferation and induce apoptosis in myeloma cells by activating the Notch3-p53 signaling pathway, highlighting the potential of SC-based compounds as promising anticancer agents [24]. Furthermore, SC was reported to inhibit miR-21 synthesis and activity by targeting the Dicer enzyme, suggesting a potential therapeutic strategy for head and neck cancer [25]. In addition, sophoridine, another quinolizidine alkaloid isolated from *Sophora flavescens*, and its structural derivatives have been shown to activate apoptotic and autophagic pathways in TNBC by suppressing mTOR signaling, thereby highlighting the plant’s broad-spectrum anticancer potential [26].

However, the antitumor effects of SC in breast cancer and its underlying molecular mechanisms remain poorly understood. We hypothesized that SC suppresses breast cancer progression by disrupting a dynamic feedback loop between oncogenic microRNAs and intracellular survival signaling cascades. To address this gap, the main objective of this study was to evaluate the antitumor activity of SC in breast cancer cell lines by examining its effects on cell proliferation, apoptosis, cell-cycle distribution, and migratory capacity. In addition, we aimed to elucidate the molecular mechanism of SC action by defining the role of miR-21 and its reciprocal regulation with the hyperactivated AKT/mTOR and ERK1/2 signaling pathways, together with their relevant downstream molecular targets.

## 2. Results

### 2.1. SC Induces Cytotoxicity and Inhibits the Survival of Breast Cancer Cells

The inhibitory effect of SC on breast cancer cell viability was preliminarily evaluated using the MTT assay. The cell viability of both types of cells showed an SC-dose-dependent reduction in cell viability (Figure 1A). The calculated IC_50_ values were 2.9 for MDA-MB-231 cells and 2.4 mM for T-47D cells. MTT assay of SC also demonstrated no toxic effect of SC on normal corneal fibroblast cells upon treatment for 48 h with the tested doses (Figure 1B).

The reduction in breast cancer cell viability was further supported by the images obtained from the light microscope; SC-treated cells showed morphological changes and a decline in the number of adherent cells at SC concentrations of IC_50_ compared to untreated cells (Figure 1C). Based on these findings, the IC_50_ values of SC were determined to be 2.9 mM for MDA-MB-231 and 2.4 mM for T-47D cells, and the corresponding ½ IC_50_ values, 1.46 mM for MDA-MB-231 and 1.2 mM for T-47D cells, were selected for subsequent experiments. To investigate more about how SC affects cancer cell development, the colony formation assay was tested on both cell lines (Figure 1D). SC significantly suppressed colony formation in a dose-dependent manner.

In MDA-MB-231 cells, treatment with SC resulted in a reduction in colony formation to 77.83% ± 3.21, 59.74% ± 5.29, and 12.53% ± 1.00 at concentrations of 0.5, 0.73, and 1.46 mM, respectively, compared to the untreated control. In addition, T-47D cells demonstrated a significantly higher reduction (*p* ≤ 0.001) in the percentage of colony formation to 60.76 ± 3.50% and 28.86% ± 2.00 following treatment with SC at doses of 0.3 and 0.6 mM. Interestingly, treatment with ½ IC_50_ (1.2 mM) caused an intense decline in colony formation to approximately 0.11% ± 0.57compared to untreated T-47D cells, with no observable colonies formed at IC_50_ doses in both cell lines.

### 2.2. SC Suppresses the Migration Ability of Breast Cancer Cells

The effect of SC on the migration ability of breast cancer cells was tested using a wound-healing assay. The results demonstrated that SC inhibited the migration rate and wound closure of breast cancer cells in a manner that is dependent on the dosage. In MDA-MB-231 cells, the percentages of wound closure after 24 and 48 h reached 11.3% ± 0.93 and 15.2% ± 1.01 at a concentration of 1.46 mM of SC, while the dose of 2.9 mM showed smaller healing areas, reaching 2.68% ± 2.22 and 5.8% ± 3.50, respectively, compared to the untreated group (49.42% ± 2.14, and 97.08% ± 0.54 after 24 and 48 h, respectively). In T-47D cells, the percentages reached after 24 and 48 h were 11.14% ± 3.54 and 15.4% ± 2.32 at the concentration of 1.2 mM of SC, and 3.33% ± 0.85 and 9.08% ± 0.31 at the concentration of 2.4 mM of SC, when compared with their respective untreated groups (66.14% ± 0.62 after 24 h and 98.08% ± 0.05 after 48 h) (Figure 1E,F). Normalization of migration data against relative cell viability suggested a concentration-dependent inhibition of motility per viable cell in both MDA-MB-231 and T-47D lines (Table S1). These results point toward potential inhibitory activities of SC on tumor cell proliferation and motility.

### 2.3. SC Induces Cell-Cycle Arrest in Breast Cancer Cells

Cell-cycle arrest and apoptosis are two major mechanisms through which cancer cell proliferation can be inhibited. To establish whether SC influenced cell- cycle progression, the distribution of SC-treated MDA-MB-231 and T-47D cells across the different cell- cycle stages was determined using flow cytometry (Figure 1G). The cell cycle analysis of MDA-MB-231 cells treated with SC concentrations (1.46 and 2.9 mM) revealed a higher percentage of cells that increased to 68.9% ± 2.05 and 85.8% ± 1.06, respectively, in the G1 phase compared to 57.6 ± 1.12% in untreated cells. This was accompanied by a decrease in the number of cells in the S and G2 phases. However, SC treatment of T-47D showed a gradual increase in cell population in the G2 phase, with a significant increase at the IC_50_ dose (2.4 mM) to 18% ± 0.61 compared to 11.12% ± 0.89 in untreated cells, with decreasing cell populations in the G1 and S phases. These results indicated that SC is capable of inducing cell cycle arrest, according to the cell type, at the G1 phase in MDA-MB-231 cells and the G2 phase in T-47D cells. One upstream step that results in apoptosis is the reduction in cell cycle progression.

### 2.4. SC Induces Apoptosis in Breast Cancer Cell Lines

Annexin V/PI labelling was used to assess the apoptotic activity of SC, and flow cytometry was used to analyse the cells. The characteristic dot plots in Figure 1H show the percentage of apoptotic cells after SC treatment in both cell lines. The percentage of apoptotic MDA-MB-231 cells increased significantly from 3.12% ± 0.17 in untreated cells to 11.06% ± 0.36 and 29.14% ± 0.68 after SC treatment at the corresponding concentrations of ½ IC_50_ and IC_50_, respectively. In SC-treated T-47D cells, the percentage was elevated from 5.44% ± 0.47 in untreated cells to 17.3% ± 0.13 at a dose of ½ IC_50_, reaching the highest increase of 42.64% ± 0.41 at a dose of IC_50_. As seen in Figure 1C, SC-treated cells exhibited remarkable morphological alterations, including reduced cell and nuclear size, nuclear condensation, and a significant loss of cell-to-cell adhesion. Such alterations are characteristic features of apoptotic cell death and became clearly evident after 48 h of SC incubation, whereas untreated cells retained their normal morphology.

### 2.5. SC Inhibits miR-21 Expression and Upregulates Its Target Genes in Breast Cancer Cells

Accumulating evidence suggests that natural agents exert their antitumor activity via multiple mechanisms, one of which is by modulating miRNA expression. So, we tried to figure out whether miR-21, one of the major oncomirs that regulate AKT/mTOR and ERK1/2 signaling in breast cancer, could be involved in the response of SC-treated tumor cells. Real-time PCR was performed to examine miR-21 expression in SC-treated and untreated breast cancer cells. Results in Figure 2A indicated that SC treatment significantly reduced miR-21 expression in MDA-MB-231 cells. Relative miR-21 expression decreased to approximately 0.35 ± 0.015 and 0.25 ± 0.057-fold following treatment with 1.4 and 2.9 mM SC, respectively, compared with untreated control cells. Notably, T-47D cells exhibited a more pronounced reduction in miR-21 expression after SC exposure, with expression levels decreasing to 0.17 ± 0.013 and 0.033 ± 0.004-fold following treatment with 1.2 and 2.4 mM SC, respectively, compared with untreated cells. These results indicate that SC effectively downregulates miR-21 expression in a dose-dependent manner in both cell lines, with a stronger suppressive effect in T-47D cells than in MDA-MB-231 cells.

Bioinformatic target analysis predicted possible miR-21 binding sites in the 3′ UTR of the human PTEN and PDCD4 transcripts, suggesting that these genes may be regulated by miR-21 (Figure 2B). Therefore, the expression levels of PTEN and PDCD4 were further investigated following SC treatment. Results of RT-PCR analysis, together with Western blotting results described later, revealed that SC increased the expression of PTEN and PDCD4 in a dose-dependent manner in both cell lines.

Treatment with SC at concentrations of ½ IC_50_ and IC_50_ significantly upregulated PTEN mRNA expression to 3.89 ± 0.11 and 7.54 ± 0.92-fold in MDA-MB-231 cells, and to 2.82 ± 0.54 and 6.45-fold in T-47D cells, respectively, compared to untreated cells (Figure 2C). At doses of ½ IC_50_, SC treatment also markedly increased PDCD4 mRNA expression by approximately three-fold in both cell lines. At the IC_50_ concentration, SC further increased PDCD4 mRNA expression by 5.47 ± 1.15- fold in MDA-MB-231 cells and 7.05 ± 1.00-fold in T-47D cells in comparison to untreated cells (Figure 2C).

Due to the important role of miR-21 in regulating intracellular signaling pathways by suppression of their negative regulators, we confirmed the positive impact of SC on PTEN protein expression by immunofluorescence staining in both cell lines (Figure 2D,E). SC- treated cells showed a marked increase in intracellular PTEN fluorescence intensity compared to untreated control cells, confirming the upregulation of PTEN expression. These findings suggest that restoration of PTEN expression may contribute, at least in part, to the tumor growth-inhibitory mechanism of SC in breast cancer cells.

### 2.6. Effect of SC on PTEN and PDCD4 Transcription After Transient Overexpression of miR-21

Based on the inhibitory effect of SC on miR-21 expression, as shown in Figure 2. Both breast cancer cell lines were transfected with miR-21 mimics. This aimed to observe whether SC regulates PTEN and PDCD4 expression via modulation of miR-21. Our findings showed that forced expression of miR-21 markedly reduced the levels of PTEN and PDCD4 transcripts to 0.026 ± 0.007 and 0.56 ± 0.015-fold, respectively, in MDA-MB-231 cells, and to 0.45± 0.01 and 0.28 ± 0.02-fold, respectively, in T-47D cells, compared with untreated cells.

Moreover, co-treatment with miR-21 mimics effectively reversed the effects of SC, restoring PTEN and PDCD4 transcript levels to baseline, achieving a non-significant change compared with MDA-MB-231 and T-47D untreated cells or scrambled mimics (Figure 3). These findings indicate that SC treatment was associated with suppression of miR-21 and restoration of PTEN and PDCD4 expression, providing molecular-level support for the involvement of the miR-21-associated regulatory axis.

### 2.7. AKT/mTOR and ERK1/2 Signaling Are Involved in SC-Mediated Tumor Inhibition and Apoptosis

Western blot analysis was carried out to evaluate the effect of SC on AKT/mTOR and ERK1/2 signaling in breast cancer cells (Figure 4A). The results of Western blotting illustrated that SC treatment significantly inhibited phosphorylation of AKT at doses of IC_50_ and ½ IC_50_ in MDA-MB-231 and T-47D cells compared with untreated controls (Figure 4B). As for the downstream signaling protein, mTOR, SC significantly decreased mTOR phosphorylation at the IC_50_ dose in MDA-MB-231 cells (Figure 4C). In T-47D cells, SC exhibited a more pronounced suppression of phosphorylated mTOR at both the IC_50_ and ½ IC_50_ doses. In addition, SC markedly inhibited ERK1/2 phosphorylation in a dose-dependent manner in both breast cancer cell lines (Figure 4D).

Western blot analysis of PTEN and PDCD4 confirmed the results of RT-PCR. PTEN protein levels were increased significantly in comparison to untreated cells, in a dose-dependent way in SC-treated cells (Figure 4E). Similarly, PDCD4 protein levels were elevated after SC treatment, with a sharp increase at the IC_50_ dose in MDA-MB-231 cells and significant elevations at both the IC_50_ and ½ IC_50_ doses in T-47D cells compared to untreated cells (Figure 4F).

To confirm the pro-apoptotic activity of SC in breast cancer cells, we evaluated caspase-3 activation by western blotting. SC treatment induced a dose-dependent increase in the protein levels of procaspase-3 and cleaved caspase-3 in both MDA-MB-231 and T-47D cells (Figure 4G,H). Moreover, expression of key genes related to cell survival and apoptosis, such as Bcl2, Bad, Bax, and caspases 8, 9, and 3, was analysed using RT-PCR analysis. SC treatment enhanced the transcription of pro-apoptotic genes Bax and Bad and reduced the expression of the pro-survival marker Bcl2 in a concentration-dependent manner (Figure 4I–M). Interestingly, SC-treated T-47D cells demonstrated a highly significant increase in the Bax/Bcl2 mRNA ratio to about 7.14 ± 0.53-fold at 1.2 mM SC and about 12.47± 1.82-fold at 2.4 mM SC, while SC treatment at the IC_50_ dose in MDA-MB-231 cells achieved a significant increase to about 4-fold, indicating a higher sensitivity of T-47D cells to SC doses. Our data showed the significant upregulation of gene expression of caspases 9, 8, and 3 gradually with increasing SC concentrations in both cell lines. These results are compatible with the flow cytometry analysis measuring the apoptotic population after SC treatment. Together, SC treatment has a profound effect on inducing apoptotic cell death in breast cancer cell lines.

### 2.8. Effect of SC on lncRNA GAS5 and HIF-1α as Downstream Mediators of AKT/mTOR and ERK1/2 Signaling

Because SC modulates AKT/mTOR and ERK1/2 signaling, we investigated its effects on two related downstream mediators, hypoxia-inducible factor-1 alpha (HIF-1α) and the long noncoding RNA GAS5, using qRT-PCR in breast cancer cells.

Our findings showed the effective downregulation of HIF-1α by SC treatment in a dose-dependent manner (Figure 5A). In MDA-MB-231 cells, HIF-1α transcript levels were significantly decreased by 25% and 49% following treatment with 1.46 and 2.9 mM SC, respectively, compared with untreated controls. In T-47D cells, HIF-1α expression showed a stronger decrease of 51% and 75% with 1.2 and 2.4 mM SC, respectively.

In addition, the outcomes demonstrated, for the first time, the profound effect of SC on lncRNA GAS5 expression in breast cancer cell lines for the first time (Figure 5B). In MDA-MB-231 cells, GAS5 expression increased to 2.95 ± 0.21-fold and 6.92 ± 0.71 -fold following treatment with 1.46 and 2.9 mM SC, respectively. In T-47D cells, GAS5 expression increased to 4.89 ± 0.29-fold at 1.2 mM SC and to approximately 8.70 ± 0.78-fold at 2.40 mM SC compared with untreated controls.

### 2.9. Effect of SC on miR196-a-5p Expression

Previous literature has demonstrated that miR-21-5p and miR196-a-5p are major targets for lncRNA GAS5 in breast cancer cells, so we also studied the effect of SC on miR196-a-5p expression in both cell lines (Figure 5C). miR-196-a-5p expression was significantly downregulated in MDA-MB-231 and T-47D cells by 57% and 73% after exposure to 1.46 mM and 1.2 mM of SC, respectively. At the corresponding IC_50_ concentrations of 2.93 and 2.40 mM, miR-196a-5p expression revealed a sharp decrease of 87% and 88%, respectively, compared with untreated controls. Overall, SC increased GAS5 expression while decreasing HIF-1α, miR-21-5p, and miR-196a-5p expression in both breast cancer cell lines.

### 2.10. Molecular Docking and Predicted Binding Modes of SC with Pre-miR-21

Molecular docking is a widely used computational approach for generating structural hypotheses regarding potential ligand–receptor interaction modes and relative binding energies within target binding pockets. Therefore, we performed docking analysis using the UTZ5 PDB structure (G22-C52) to investigate the potential binding of SC and streptomycin to the miR-21 precursor. Notably, UTZ5 does not fully represent the native structure of pre-miR-21 (G8 to U66) during Dicer recognition. Nevertheless, it includes the nucleotide region containing the Dicer cleavage site (between A29 and C46).

Docking energy levels below −4.25 kcal/mol are generally considered to show some binding activity between the two molecules, whereas those below −5.0 kcal/mol are considered to suggest good binding activity, and those below −7.0 kcal/mol indicate strong binding activity [27]. In this study, the blind docking process showed that SC could bind to the miR-21 precursor in two sites, including the terminal loop and near the Dicer cleavage site, as shown in Figure 6A. The binding mode of SC exhibited an energy binding of −7.22 kcal/mol against the microRNA-21 precursor terminal loop, forming five hydrophobic interactions with G35, U34, and U38, in addition to forming two hydrogen bonds with U33, and C39 (Figure 6B–D). Interestingly, the docking research revealed another binding location (Figure 6E–G): at the Dicer cleavage site, demonstrating an energy binding of −5.8 kcal/mol, interacted with A29 by one hydrogen bond and U43 by π-cationic interaction (electrostatic contact). It also established two hydrophobic π-alkyl contacts with G44 and G45. In addition, there was another binding mode of SC (binding mode 4), which exhibited a binding energy of −5.6 kcal/mol against the miR-21 precursor dicer cleavage site (Figure 6H). This mode showed that SC interacted with G28 and U43 by two conventional hydrogen bonds. SC also formed π-alkyl and π-sigma interactions with U43 and G45 (Figure 6I,J). Analysis of bonds in SC-pre-miR-21 interactions is shown in Table 1. This was comparable to streptomycin, a previously reported pre-miR-21-binding aminoglycoside, which also showed favorable binding to both regions of pre-miR-21, but with a lower predicted affinity than SC in the terminal loop. Streptomycin exhibited a binding energy of −6.47 kcal/mol and formed seven conventional hydrogen bonds with U33, U34, G35, U38, C39, and U40, with an average bond length of 2.48 Å (Figure 7A–D). At the Dicer cleavage site, streptomycin showed a stronger interaction, with a binding energy of −7.5 kcal/mol. In this mode, streptomycin formed ten hydrogen bonds with U26, U27, U31, C30, G44, and G45, with an average bond length of 2.60 Å. In addition, it formed two electrostatic interactions with U27 and G28, as well as π-sulfur interactions with A29 (Figure 7E–H). Analysis of bonds in Streptomycin-pre-miR-21 interactions is shown in Table 2. Overall, SC demonstrated a more favorable interaction profile than streptomycin in the terminal loop region, while streptomycin showed stronger binding at the Dicer cleavage site. According to these results, SC might be a useful structural scaffold for binding pre-miR-21 and influencing its processing.

## 3. Discussion

Natural products represent promising therapeutic candidates for cancer therapy due to their multi-target capabilities and lower toxicity than conventional anticancer agents. Sophocarpine (SC), a plant-derived alkaloid, exhibits broad antitumor activity in several cancer models, but its mechanisms in breast cancer remain unknown. The present study provides evidence that SC suppresses breast cancer cell growth by downregulating miR-21 and concurrently inhibiting the AKT/mTOR and ERK1/2 signaling pathways, revealing a mechanistic network that links miR-21 suppression to dual-pathway blockade. SC treatment revealed a cytotoxic effect on two biologically distinct breast cancer cell lines: MDA-MB-231 cells, a poorly differentiated and highly invasive TNBC cell line, and T-47D, a well-differentiated ER+, PR+ breast cancer cell line. In both cell lines, SC suppressed cell proliferation, impeded cell migration and cell cycle progression, and induced apoptosis. These results indicated that SC exerts anti-proliferative and pro-apoptotic effects on breast cancer cells. SC inhibited the proliferation of MDA-MB-231 and T-47D breast cancer cell lines in a dose-dependent manner. Although the effective concentrations were relatively high (millimolar range), SC showed no significant cytotoxicity toward normal corneal stromal fibroblast cells under the same experimental conditions, which serves as a preliminary evaluation of general cellular tolerance in primary non-transformed cells. Similar concentration ranges of SC have also been reported in studies involving other cancer cell types [28,29,30]. These results are consistent with previous studies in which SC had no toxicity on H9c2 cardiomyoblasts at concentrations up to 10 mM or on normal human astrocytes [29,31]. In addition, previous in vivo studies reported no obvious organ toxicity associated with SC administration under the specific experimental conditions examined [22]. However, from a translational standpoint, it is important to acknowledge that the observed IC_50_ values for SC (2.9 mM and 2.4 mM) fall within the millimolar range, indicating limited direct translational relevance in their native form. Accordingly, these findings suggest that SC may serve as a potential structural scaffold or pharmacological tool compound. For context, clinically established chemotherapeutic agents such as doxorubicin generally exhibit IC_50_ values in the nanomolar-to-micromolar range in breast cancer cell lines. Under broadly comparable 48 h MTT conditions [32], Wen et al. reported IC_50_ values of approximately 3.16 µM for MDA-MB-231 cells and 8.53 µM for T-47D cells. These values were lower than the IC_50_ values observed for SC in the present study, highlighting that SC may require further structural optimization to enhance its activity.

Available pharmacokinetic evidence, derived predominantly from animal studies, suggests that sophocarpine is rapidly absorbed, broadly distributed, and comparatively rapidly eliminated [21]. However, absolute oral bioavailability, human plasma exposure, tumor-tissue concentrations, and the relationship between systemic exposure and toxicity remain insufficiently characterized [21]. Therefore, it is currently unclear whether the millimolar concentrations observed in vitro can be achieved at the tumor site without causing unacceptable systemic toxicity.

Future work must focus on medicinal chemistry strategies—such as core functionalization or chemical derivatization—to improve potency into the micromolar or nanomolar range. Nanoparticle-based delivery systems may also be explored to improve solubility, stability, pharmacokinetic exposure, biodistribution, and localized tumor accumulation. However, such approaches would require independent evaluation of loading efficiency, release kinetics, pharmacokinetics, biodistribution, efficacy, and toxicity.

The clonogenic assay further demonstrated that the inhibitory effects of SC persisted after its withdrawal, indicating sustained suppression of the reproductive capacity of breast cancer cells. This finding is consistent with previous observations in other cancer models [22,28].

Hyperactivation of AKT/mTOR and ERK1/2 pathways drives breast cancer proliferation, survival, and therapy resistance through extensive cross-talk. Activated AKT inhibits the TSC complex, thereby activating mTORC1 and promoting proliferative and survival signaling in cancer cells [33]. ERK1/2 activation is common in breast cancer. Activated ER binds and directly activates RAS, while RTK activation stimulates the Grb2/Shc/Sos complex, leading to activation of the RAS/ERK pathway [34]. Activated ERK1/2 triggers phosphorylation events and induces transcription of genes controlling survival, proliferation, and migration [5]. In the present study, SC directly inhibited AKT phosphorylation at Ser473, followed by mTORC1 phosphorylation at Ser2448 in both cell lines. These results are consistent with previous reports demonstrating inhibition of AKT/mTOR signaling by SC in prostate cancer and glioblastoma models [22,29]. SC also reduced ERK1/2 phosphorylation dose-dependently in both breast cancer cell lines. This result agrees with previous studies showing that SC inhibited the phosphorylation of ERK1/2 in colorectal cancer cells and in experimental models of liver fibrosis [35,36]. The concurrent suppression of AKT/mTOR and ERK1/2 signaling is mechanistically important because these pathways participate in regulatory interactions. AKT regulates ERK1/2 activity through PKC signaling, whereas mTORC1 can enhance RAS/ERK signaling through S6K-mediated feedback regulation [37,38]. Accordingly, inhibition of AKT phosphorylation by SC may contribute indirectly to reduced ERK1/2 activation. Conversely, ERK1/2 and its downstream kinase RSK can stimulate mTORC1 activity by phosphorylating TSC2 and PRAS40 and relieving their inhibitory effects on mTORC1 [39,40]. This explains why SC’s ERK1/2 inhibition may contribute to mTORC1 suppression at Ser2448, particularly in T-47D cells where cross-activation between pathways is prominent [41,42]. This dual inhibition breaks reciprocal positive feedback loops that typically drive resistance, providing evidence for SC efficacy as an anticancer drug.

MicroRNAs serve as critical diagnostic biomarkers and therapeutic targets due to their capacity to orchestrate tumor progression. Accumulating evidence indicates that natural compounds can modulate miRNA expression profiles, helping explain how these compounds counteract treatment resistance [43]. miR-21 is the most overexpressed oncomir in breast cancer, promoting growth, migration, and metastasis by targeting tumor suppressors [9]. Overexpression of miR-21 was reported in diverse types of breast cancer cell lines, such as MCF-7, T-47D, and MDA-MB-231 cells [44,45]. In breast cancer cells, miR-21 expression is up-regulated via different signaling pathways such as AKT/mTOR and MAPK/ERK1/2 signaling through transcriptional and post-transcriptional mechanisms, creating positive feedback loops [45]. In this study, SC downregulated miR-21 expression levels in both breast cancer cell lines in a dose-dependent manner.

Our results showed that SC suppressed miR-21 potentially via interconnected regulatory mechanisms: First, SC may interfere directly with Dicer-mediated pre-miR-21 processing. Dicer cleavage of pre-miR-21 is an essential step in the generation of mature miR-21. Targeting the stem-loop structures of pri-miRNAs or pre-miRNAs has been proposed as a strategy for selectively reducing mature oncogenic miRNAs in disease [46,47]. Molecular docking analysis provided a plausible dual-site binding model for SC—the terminal loop (higher affinity) and near the Dicer cleavage site—with interactions including U43, G44, G45, and hydrogen bonds with A29 (mode 2) and G28 (mode 4). Based on the literature, Dicer cleaves pre-miR-21 between A29-C46 at residues G28-A29-C30 and G45-C46 [48,49]. This computationally predicted overlap suggests that SC binding may physically interrupt Dicer access, providing a structural basis for the observed reduction in mature miR-21 levels in breast cancer cells. SC binds with higher affinity to the terminal loop, which is large, flexible, and devoid of stable base pairs, providing a binding site for RNA-binding regulatory proteins [49]. The terminal loop is critical for Dicer binding affinity and cleavage activity [50,51]: The N-terminal DExH/D helicase domain of Dicer engages the terminal loop to correctly position pre-miRNA for precise RNase III cleavage [51,52]. Furthermore, the flexible loop facilitates Dicer processing by enhancing enzyme turnover on RNA substrates [53]. Therefore, SC binding to the terminal loop may reduce loop flexibility and/or Dicer RNase domain binding affinity, leading to imprecise cleavage and decreased mature miR-21 levels. Although SC exhibited a higher affinity for the terminal loop, the stability of the predicted SC–pre-miR-21 binding poses requires further validation through molecular dynamics simulations and experimental RNA-binding assays. The results align with a previous study showing SC downregulated miR-21 by blocking pre-miR-21 Dicer processing in vitro [25].

Streptomycin is an aminoglycoside, previously reported as a specific inhibitor of miR-21, due to its ability to bind to pre-miR-21 at a region close to the terminal loop [54]. Previous reports established that streptomycin downregulates miR-21 by binding to pre-miR-21 and blocking the function of the Dicer enzyme, an essential step in miRNA maturation [54,55]. Comparative docking with streptomycin in this study showed a lower predicted affinity than SC at the terminal loop, yet showed stronger binding at the Dicer cleavage site, where it formed extensive hydrogen-bonding and electrostatic interactions. This pattern may be explained by the structural nature of streptomycin as a highly cationic aminoglycoside, which binds structured RNA through multivalent electrostatic interactions with the phosphate backbone and base edges, particularly at the stem-loop junction and bulged regions of pre-miRNA [54,56].

Notably, SC and streptomycin both engage the same critical residues (A29, G28, G44, G45) within the Dicer cleavage site, which is mechanistically important. The fact that SC, despite its different chemical scaffold, recapitulates binding to these key residues suggests a convergent recognition of functionally critical RNA motifs, supporting the specificity and potential functional relevance of SC as a pre-miR-21-binding ligand. However, SC’s preference for the terminal loop may be more effective at perturbing Dicer recognition and catalytic positioning. These in silico predictions serve as an exploratory hypothesis, offering a molecular rationale for how SC may modulate pre-miR-21 maturation through direct steric hindrance of Dicer cleavage-site access and perturbation of terminal-loop dynamics.

Second, HIF-1α Transcriptional Suppression: The AKT/mTOR and ERK1/2 signaling pathways augment miR-21 transcription by enhancing transcription factors that bind to the miR-21 promoter [57,58]. HIF-1α, upregulated in breast tumors, prevents apoptosis and promotes cell cycle progression, angiogenesis, migration, and drug resistance [15,59]. HIF-1α expression, stabilization, and transcriptional activation are promoted by AKT/mTOR and ERK1/2 signaling [59,60]. Moreover, MAPK/ERK1/2 activity is essential for HIF-1α upregulation even at physiological oxygen tensions in cancer cells [61,62]. HIF-1α can directly regulate miR-21 transcription by binding its promoter, establishing a positive feedback loop between HIF-1α and miR-21 [63]. In this context, our data demonstrated that SC significantly reduced HIF-1α gene expression levels dose-dependently in both breast cancer cell lines. This contributes to miR-21 reduction by decreasing transcription following AKT/mTORC1 and ERK1/2 suppression by SC. In previous reports, SC has been reported to inhibit the tumorigenesis of colorectal cancer by downregulation of the MEK/ERK pathway and inhibiting VEGF expression, a well-known HIF-1α target [35]. Third, lncRNA GAS5 Upregulation: SC may regulate miR-21 through upregulation of the tumor-suppressive lncRNA GAS5. Activation of mTOR can enhance miR-21 levels through a post-transcriptional mechanism by regulating lncRNA GAS5. GAS5 is a putative tumor-suppressive lncRNA that is frequently expressed at low levels in breast cancer [14]. It promotes apoptosis and cell- cycle arrest while inhibiting tumor cell growth [64,65]. Because of the small ORF and several termination codons in its sequence, high mTOR activity encourages GAS5 degradation via the nonsense-mediated decay (NMD) pathway. On the other hand, the translation of the GAS5 short ORF is hindered when the mTOR pathway is inactivated, as in growth-inhibited cells. This prevents NMD and raises the amounts of GAS5 transcripts [66,67]. Furthermore, it was also suggested that GAS5 short ORFs could “collect” ribosomes on themselves, acting as a “sponge” for ribosomes, resulting in translation inhibition of oncogenic proteins [68]. Inhibition of the AKT/mTOR pathway has been shown to enhance GAS5 levels in breast cancer cells [69]. Results of the current study showed that, for the first time, SC significantly augmented GAS5 transcript levels dose-dependently in both cell lines. This increase is consistent with the inverse relationship between GAS5 and mTOR activity. The negative correlation between GAS5 expression and miR-21 levels has been observed in both breast tumor specimens and cell lines. Previous studies have indicated a reciprocal regulatory relationship between miR-21-5p and GAS5 in breast cancer cells [70]. This regulation is attributed to the ability of GAS5 to act as a molecular sponge for miR-21 via miRNA response elements located in exon 4 of the GAS5 transcript [71,72]. In this study, SC inhibited mTOR, leading to GAS5 elevation, which sponges miR-21 and reduces its availability in breast cancer cells.

The present results also showed that SC significantly downregulated miR-196a-5p expression dose-dependently in T-47D and MDA-MB-231 cells. This effect may be associated with increased GAS5 expression because GAS5 has been reported to bind and negatively regulate miR-196a-5p. Negative correlations between GAS5 and miR-196a-5p have been described in several cancer types [73,74]. In breast cancer, miR-196a-5p has oncogenic functions. Its transcription may be regulated by Erα, and increased miR-196a-5p expression has been observed in triple-negative breast cancer tissues, where it is associated with aggressive behaviour [75,76]. miR-196a-5p promotes AKT signaling by targeting FOXO1 and/or p27, thereby promoting cell-cycle progression, proliferation, and apoptosis inhibition [77,78,79]. Conversely, its downregulation decreases T-47D and MCF-7 cell proliferation in vitro and suppresses tumor growth in vivo [80]. In this study, SC-induced lncRNA GAS5 upregulation and miR-196a-5p downregulation as novel breast cancer-associated observations suggest a potential lncRNA–miRNA regulatory mechanism. In support of the present findings, overexpression of GAS5 in MDA-MB-231 cells significantly suppressed both miR-21-5p and miR-196a-5p, thereby impairing downstream AKT phosphorylation [76]. Within this ceRNA network, SC activates lncRNA GAS5, which sponges both miRNAs, resulting in direct AKT/mTOR pathway suppression via targeting tumor suppressor genes.

MiR-21 regulates the AKT/mTOR pathway by targeting tumor suppressors such as PTEN and PDCD4, which play essential roles in the regulation of the cell cycle, induction of apoptosis, and growth inhibition [81,82]. The present data from TargetScan prediction confirmed the potential binding of miR-21 to the 3 ′ UTR of PTEN and PDCD4 mRNA as previously reported in several studies, indicating their importance as functional targets of miR-21 in breast cancer cells [82,83].

By dephosphorylating PIP3, which lowers AKT phosphorylation and mTOR activation, PTEN, the primary negative regulator of the PI3K/AKT pathway, opposes PI3K activity [38,84]. PTEN is abnormally downregulated in breast cancer, and loss of PTEN expression is associated with poor prognosis [85]. PTEN also inhibits ERK1/2 by dephosphorylating Shc, preventing Grb-SOS recruitment to the plasma membrane, and blocking Ras/Raf/ERK1/2 cascade activation [86]. miR-21 inhibits PTEN, while activated AKT and ERK further reduce PTEN transcription, creating a reciprocal regulatory loop [87,88].

PDCD4 is another direct target of miR-21, and its suppression contributes to cancer cell invasion and metastasis [89]. PDCD4 regulates cell growth, proliferation, and motility by affecting the AKT/mTOR pathway [81]. It inhibits translation initiation by blocking eIF4A activity, suppressing oncogenic protein translation, and binding to 5′ mRNA internal ribosome entry sites to suppress anti-apoptotic proteins like Bcl-xl, inhibiting breast cancer progression [13]. PDCD4 also inhibits AKT activation by suppressing Rictor (a crucial mTORC2 component) and suppresses AP-1-mediated transactivation that participates in miR-21 transcription through AKT activation [82,90]. Additionally, mTORC1 and ERK are key regulators of PDCD4 abundance; activated mTOR and ERK1/2 promote PDCD4 proteasomal degradation in cancer cells [91,92]. Previous reports have shown that PDCD4 overexpression reduced the rate of proliferation and promoted apoptosis in T-47D, MDA-MB-231, and MCF-7 breast cancer cells [93,94].

In the present study, as anticipated, increasing concentrations of SC led to a significant upregulation of both mRNA and protein levels of PTEN and PDCD4 in breast cancer cell lines. Moreover, the great impact of SC on PTEN activation was supported by immunofluorescence staining. These findings agree with previous results showing that SC upregulated PTEN and PDCD4 by inhibiting miR-21, resulting in the inhibition of cell growth in glioblastoma cells and head and neck cancer cells [25,28,29]. Other natural compounds have similarly been shown to interact with pre-miR-21 or reduce mature miR-21 expression, resulting in restoration of PTEN and PDCD4 and suppression of tumor growth [10,95,96].

The miR-21 mimic experiments provided additional molecular-level support for the involvement of miR-21 in the effect of SC. Forced miR-21 expression reduced PTEN and PDCD4 transcript levels, whereas combined miR-21 mimic transfection and SC treatment attenuated the SC-induced increases in PTEN and PDCD4. This result is consistent with previous findings in glioblastoma cells [29]. Moreover, overexpression of miR-21 by mimics reversed the effect of SC and restored cell proliferation, invasion, and metastasis in head and neck cancer [25]. These data confirm that increased PTEN and PDCD4 transcript levels resulted directly from SC-mediated suppression of miR-21, establishing SC as a potential small-molecule inhibitor of miR-21.

Numerous studies confirm the correlation between miR-21, HIF-1α, and PTEN signaling in breast cancer [97,98]. HIF-1α expression is regulated by PTEN, indicating that SC’s effect on HIF-1α is linked to PTEN upregulation. Additionally, our data align with studies showing that GAS5 transcripts are associated with PTEN and PDCD4 expression in breast cancer via miR-21 molecular sponging [69,99]. GAS5 overexpression has been shown to enhance apoptosis by increasing PTEN and reducing p-AKT through miR-21 modulation [100,101], confirming that SC activates the GAS5/miR-21/PTEN axis to suppress AKT/mTOR signaling and promote tumor cell apoptosis. These results support miRNA-based targeting of signaling pathways as a key strategy to combat breast cancer. Collectively, these findings highlight the significant role of SC in modulating the miR-21/PTEN/AKT/ERK1/2 signaling axis, suggesting this pathway as a key mechanism underlying SC’s anti-tumor effects in breast cancer.

In cancer, the evasion of cell death is a critical mechanism by which abnormal cells become malignant [102]. Targeting pathways that regulate apoptosis and cellular proliferation is a key strategy in cancer therapy. Based on our findings, SC appears to induce apoptosis by suppressing miR-21 and modulating the AKT/mTOR and ERK1/2 signaling pathways. The Bcl-2 protein family is essential for controlling cell death. Pro-apoptotic members like Bax and Bad, located on the mitochondrial membrane, promote intrinsic apoptosis by releasing cytochrome c, which triggers caspase activation [103,104]. Releasing cytochrome c facilitates the dimerization and activation of caspase 9, subsequent cleavage, and activation of effector caspase 3. Activated caspase 8 also promotes caspase 3 cleavage through activation of pro-apoptotic Bid, which can activate the Bax protein [105]. Caspase 3 facilitates the targeted cleavage of numerous crucial cellular proteins, which in turn triggers the apoptotic process [106]. Bcl-2 can inhibit this process by forming heterodimers with Bax, thereby preventing cell death [103,107]. However, proteins like Bad and Bim can bind to and inhibit Bcl-2, promoting apoptosis [108]. In breast cancer, AKT/mTOR and ERK1/2 signaling shift the balance of Bcl-2 family proteins toward survival by suppressing pro-apoptotic proteins and promoting anti-apoptotic signaling [109,110]. SC treatment upregulated pro-apoptotic genes Bax and Bad while downregulating Bcl-2 in breast cancer cells. This shift in the Bax/Bcl-2 ratio appeared highly significant in T-47D cells, facilitating cytochrome C release, increasing caspase-3 levels, and leading to apoptosis. SC significantly upregulated caspase-8, caspase-9, and caspase-3 transcription levels dose-dependently. Western blot analysis confirmed caspase-3 activation through increased levels of cleaved caspase-3. These molecular results were in line with an Annexin V/PI flow cytometry analysis that revealed a concentration-dependent rise in apoptotic cells, with T-47D cells showing higher sensitivity to SC. SC-treated cells also displayed characteristic apoptotic morphology, including cell shrinkage, rounding, nuclear condensation, and detachment (Figure 1C). These changes aligned with established hallmarks of apoptosis [111,112]. These findings were in line with other research that has shown that SC promoted apoptosis via modifying Bcl-2 family proteins and blocking the AKT/mTOR pathway [22,29]. SC and its derivatives have been shown to trigger apoptosis in various cancer types by similar mechanisms [24,113]. Suppression of miR-21 may further contribute to apoptosis through restoration of PTEN and PDCD4 and modulation of Bax and Bcl-2 expression [29,114,115,116]. Elevation of GAS5 could also promote apoptosis through activation of Bax, Bak, and caspase 3 and 9 expression in cancer cells [117]. It is worth noting that HIF-1α-mediated inhibition of apoptosis through upregulation of Bcl-2 and downregulation of Bax, Bad, and Bak is associated with increased drug resistance in breast cancer cells [61,118]. Therefore, the concurrent suppression of miR-21 and HIF-1α and restoration of GAS5, PTEN, and PDCD4 may collectively contribute to the pro-apoptotic activity of SC.

In addition to Apoptosis, the induction of cell cycle arrest is a critical mechanism in anticancer therapy. Uncontrolled cell cycle progression is a hallmark of tumor cells, driving cancer development and progression. Cyclins, checkpoints, and cyclin-dependent kinases (CDKs) govern cell cycle progression through stage-specific regulation [119]. This process is tightly controlled by the signaling pathways AKT/mTOR and ERK1/2. Activated AKT and ERK promote cell cycle advancement by phosphorylating and inhibiting CDK inhibitors while activating cyclin proteins, positively regulating the G1/S and G2/M transitions, and enhancing cell proliferation and mitotic capacity [120,121,122]. Conversely, PTEN and PDCD4 counteract these effects to prevent premature DNA replication and mitosis [123,124]. Additionally, miR-21 and miR-196a regulate cell cycle progression by targeting cell cycle regulators across various cancer types [98,125]. In the present study, our results demonstrated that SC was able to restrain MDA-MB-231 cells in the G1 phase, with a decrease in the G2 and S phase cell population in a dose-dependent manner. However, it induced arrest in the G2 phase in the T-47D cell line, with a decrease in G1 and S phases. These results agree with previous studies showing that SC treatment arrested the cell cycle in the G1 phase with inhibition of AKT activity in HCC and glioblastoma through inhibition of cyclin D1 and upregulation of P27 [29,126]. Similarly, matrine, a structurally related Sophora alkaloid, activated cell cycle regulators (P21 and P27) through the miR-21/PTEN/AKT signaling pathway in breast cancer cells, causing cell cycle arrest at the G1 phase [12]. On the other hand, inhibition of AKT/mTOR signaling has been shown to arrest the G2 phase in different breast cancer cell lines, including T-47D cells, by downregulation of G2/M accelerator cdc25 and cdc2, and upregulation of P21 [127,128].

SC also suppressed breast cancer cell migration in a concentration-dependent manner. While we acknowledge that anti-migratory and cytotoxic effects inherently overlap at these effective concentrations, normalizing migration data against relative cell viability suggested a continued suppression of motility per viable cell (Table S1). These findings provide supportive evidence of an antimigratory effect beyond the reduction in cell viability.

Based on previous reports, the observed impairment of wound closure may involve the inhibition of miR-21 and suppression of the AKT/mTOR and ERK1/2 signaling pathways, leading to modulation of their downstream targets involved in epithelial-to-mesenchymal transition (EMT) and inhibiting the migration of tumor cells [129]. EMT causes the remodeling of the cytoskeleton and the formation of lamellipodia, which can be suppressed by the inhibition of mTOR, thereby weakening the abilities of migration and invasion [130]. In addition, downstream targets of these pathways activate transcription factors to convert epithelial cells into cells with a mesenchymal phenotype [131]. A strong correlation has also been found between miR-21 and EMT, viability, migration, and invasion of breast cancer cells [132]. Consistent with previous reports, SC treatment was associated with modulation of these pathways and with changes in EMT-related markers, including reduced mesenchymal features and increased E-cadherin expression [22,25,35,123]. By targeting miR-21, SC has also been shown to inhibit the migration of head and neck cancer cells, leading to the reversal of EMT [25].

Collectively, the current findings support a model in which SC suppresses breast cancer cell growth through coordinated regulation of an interconnected miRNA-signaling network. SC reduced miR-21 expression through potentially complementary mechanisms, supported by an exploratory computational hypothesis suggesting interference with pre-miR-21 processing, alongside downregulation of HIF-1α and upregulation of GAS5.” Reduced miR-21 activity was accompanied by restoration of PTEN and PDCD4, inhibition of AKT/mTOR and ERK1/2 signaling, suppression of proliferation and migration, induction of cell-cycle arrest, and activation of caspase-dependent apoptosis. The proposed mechanism is summarized in Figure 8. Nevertheless, the molecular mechanism by which SC regulates miR-21 requires additional validation through in vivo experimental studies.

Study Limitations: Primary rat corneal stromal fibroblasts provided an initial indication of non-cancerous cell tolerance. However, testing against normal human mammary epithelial cells (e.g., MCF-10A) is still required to confirm mammary-specific selectivity and study nonspecific or off-target effects. Additionally, future cytotoxicity assays should include standard chemotherapeutic controls to benchmark relative efficacy and dose–response kinetics. Despite these limitations, the findings provide preliminary insights into the antitumor activity of SC against breast cancer cells; however, comprehensive in vivo evaluations—including animal tumor efficacy, tumor tissue accumulation, pharmacokinetics, and systemic toxicity profiles—are strictly required before any translational or clinical inferences can be drawn.

## 4. Materials and Methods

### 4.1. Reagents and Materials

SC (C15H22N2O; MW = 246.35; purity ≥ 95%) was purchased from Sigma-Aldrich (St. Louis, MO, USA). MTT (3-(4,5-dimethylthiazol-2-yl)-2,5-diphenyl-2H-tetrazolium bromide) was purchased from SERVA (Heidelberg, Germany). All SC preparations were freshly dissolved in free DMEM on the day of each experiment. Propidium Iodide (PI)/RNase Staining Solution was obtained from Cell Signaling Technology (Danvers, MA, USA). FITC Annexin V Apoptosis Detection Kit was purchased from BD Biosciences (San Jose, CA, USA). Vivantis ViPrime PLUS Taq qPCR Green Master Mix 1 was purchased from Vivantis Technologies (Subang Jaya, Malaysia). miRNeasy Mini kit, miScript II RT Kit, miScript SYBR Green PCR Kit, miR21-5p specific miScript Primer Assay, and miR196a-5p specific miScript Primer Assay were purchased from Qiagen (Hilden, Germany). The other primers were purchased from Bio-Basic (Markham, ON, Canada). AKT pS473 Rabbit polyclonal antibody was purchased from Abbexa (Cambridge, UK). Phospho-mTOR (Ser2448) Rabbit polyclonal antibody was obtained from Invitrogen (Thermo Fisher Scientific, Waltham, MA, USA). The other primary antibodies were obtained from Cell Signaling Technology (Danvers, MA, USA). Alexa Fluor 488-conjugated anti-Rabbit IgG secondary antibody and AP-linked secondary antibody anti-Rabbit IgG were purchased from Cell Signaling Technology (Danvers, MA, USA). HRP-conjugated anti-rabbit secondary antibody was obtained from Invitrogen (Thermo Fisher Scientific, Waltham, MA, USA). 1-Step™ NBT/BCIP Substrate Solution was purchased from Thermo Fisher Scientific (Waltham, MA, USA). Hoechst 33342 Staining Dye Solution was purchased from Abcam (Cambridge, UK).

### 4.2. Cell Lines

MDA-MB-231 and T-47D cell lines were obtained from the American Type Culture Collection (Manassas, VA, USA). Normal corneal stromal fibroblast cells, which were isolated from adult rats, were obtained from the Faculty of Medicine, Alexandria University, Egypt [133]. Cell lines were screened for mycoplasma infection (Look-Out Mycoplasma PCR detection kit, Cat. No. MP0035, Lot No. BCCB7403; Sigma-Aldrich, St. Louis, MO, USA). Additionally, they were routinely verified by morphology and growth curve analysis. Cells were maintained in Dulbecco’s modified Eagle’s medium (DMEM) (Lonza, Basel, Switzerland) supplemented with 10% (*v*/*v*) fetal bovine serum (FBS) (Sigma-Aldrich, St. Louis, MO, USA) and antibiotics (100 U/mL penicillin, 100 µg/mL streptomycin) (Lonza, Basel, Switzerland). The cultures were maintained at 37 °C in 5% CO_2_ at atmospheric humidity, and the media were changed every 3 days. Cells were passaged upon reaching 80–90% confluence using 0.25% (*w*/*v*) trypsin/ethylene diamine tetraacetic acid (EDTA) (Lonza, Basel, Switzerland), then were plated in T25 cm^2^ flasks or other culture vessels (6- or 96-well plates) according to the experiment conducted. Cell line investigations were conducted at the Alexandria University Faculty of Medicine’s Center of Excellence for Research in Regenerative Medicine and its Applications (CERRMA).

### 4.3. MTT Cytotoxicity Assay

Cell viability, proliferation, and cytotoxicity are all indicated by cellular metabolic activity, which is measured using the MTT assay [32]. Normal Corneal Fibroblast cell line, MDA-MB- 231, and T-47D cells were cultured till mid-log phase and seeded in a 96-well plate at a density of 7 × 10^3^ cells/well in 100 µL of complete medium for 24 h before treatment. After 24 h of adherence, the seeding medium was changed to different concentrations of SC (0.25, 0.5, 1, 1.5, 2, 2.5, 3, 3.5, 4, and 5 mM) at 37 °C under 5% CO_2_ for 48 h. After 48 h of incubation, the culture medium was aspirated and replaced with 100 µL of fresh complete medium supplemented with 10 µL of MTT reagent (5 mg/mL MTT [3-(4,5-dimethylthiazol-2-yl)-2,5-diphenyltetrazolium bromide] in sterile DPBS). Cells were then incubated for an additional 3 h at 37 °C in a humidified incubator with 5% CO_2_. After discarding the medium, all wells, including blank wells, were filled with 100 µL of DMSO. For twenty minutes, the plate was gently shaken and allowed to stand at room temperature in the dark. Finally, the absorbance was measured at 570 nm in a microtiter plate ELISA reader (Tecan, Infinite F50, Männedorf, Switzerland). The viability of cells was calculated as follows: % viability = (Absorbance of sample at 570 nm/Absorbance of untreated at 570 nm) × 100. The effect of different concentrations of SC on cell viability was used to calculate IC_50_ (the concentration required to kill 50% of the cells). Results were expressed as mean ± SE (experiments were repeated in triplicate for each concentration and compared to untreated control experiments).

### 4.4. Colony-Formation Assay

The clonogenic assay is a cell survival assay that measures the ability of a single cell to form a colony. The assay evaluates the cell’s capacity to divide with and without treatment, thereby determining the drug’s cytotoxicity [134]. A colony was defined as a cluster consisting of at least 50 cells. Cancer cells (1 × 10^3^ per well) were seeded into six-well plates. After 24 h, the medium was replaced with SC at varying concentrations for the treatment of MDA-MB-231 and T-47D cells. Treatment continued for 48 h, then the medium was refreshed every 2–3 days for a total of nine days. At the end of the treatment period, cells were washed with PBS, fixed with ice-cold methanol, washed again with PBS, and stained with 3% (*w*/*v*) Crystal Violet (Sigma-Aldrich, Burlington, MA, USA). Visible colonies were counted, and representative images were captured using a digital camera (Canon DS126211; Canon, Tokyo, Japan).

### 4.5. Cell Migration/Wound Healing Scratch Assay

The efficacy of SC in reducing the migration of cancer cells was tested using the scratch assay [135]. In order to facilitate cellular attachment, MDA-MB-231 and T-47D were plated in 6-well plates at a density of 3 × 10^5^ cells per well and incubated for a full day. A sterile 200 µL pipette tip was used to make a linear scratch. Two PBS washes were used to remove detached cells. Treated cells received SC at concentrations corresponding to 1/2 IC_50_ and IC_50_ for both cell types in serum-free (starvation) medium, while the control group received only serum-free medium. Images of the wound area were captured immediately after scratching (designated as 0 h), and subsequently at 24 and 48 h, using an inverted phase contrast microscope (Olympus CKX41SF, Tokyo, Japan). ImageJ software (Version 1.52p software 64, NIH, USA) was used to quantify the extent of the wound healing area after representative photos were captured. The percentage of wound closure was calculated as follows:

% Wound closure = (wound area at zero time-wound area at a specific time interval)/(wound area at zero time) × 100

To rule out the confounding effect of cytotoxicity on cell motility, the Normalized Migration Index was calculated by dividing percentage wound closure by relative cell viability. The calculation method and normalized values are provided in Table S1.

### 4.6. Cell Cycle Assay

PI/RNase was used as a nuclear stain in fluorescence microscopy and as a DNA content determinant in cell cycle analysis by flow cytometry [136]. MDA-MB-231 and T-47D cell lines were subjected to SC concentrations of IC_50_ and _1/2_ IC_50_ for both cell lines. Following a 48 h incubation period, the cells were trypsinized, harvested in Eppendorf tubes with ice-cold methanol for at least an hour at 4 °C, centrifuged, and the methanol was disposed of. A BD FACSCalibur flow cytometer (Becton Dickinson Biosciences, San Jose, CA, USA) was used for analysis after the cells were washed with 3 mL PBS, resuspended with 1 mL PI/RNAase staining solution, and incubated for 15 min. Cell division ratios in G1/S and G2/M phases were consequently determined using BD Cell Quest Pro Software version 5.1. The ratios were presented as the mean of three separate experiments.

### 4.7. Cell Apoptosis Assay Using a Flow Cytometer

MDA-MB-231 and T-47D cell lines were added to 6-well plates and cultured for 24 h, followed by treatment with SC concentrations of IC_50_ and 1/2 IC_50_ for both cell lines. The treatments were administered for a duration of 48 h. Cells were washed three times in PBS, trypsinized, and harvested in Eppendorf tubes for staining with PI (propidium iodide) and Annexin V-FITC (fluorescein isothiocyanate) [137]. The cell pellets were resuspended in 1 mL of 1× binding buffer after being rinsed twice with PBS and once with 1× binding buffer. Five microliters of Annexin V-FITC and five microliters of PI were added to a clean tube containing one hundred microliters of the cell solution. Samples were incubated in the dark at room temperature for fifteen minutes. 400 µL of 1× binding buffer was then added. As directed by the Annexin V-FITC kit (Becton-Dickinson, Franklin Lakes, NJ, USA), the samples were analyzed using a BD FACSCalibur flow cytometer and cell sorter with Cell QuestTM software version 5.1. The viable cells (the lower left quadrant), early-phase apoptotic cells (the lower right quadrant), late-phase apoptotic or dead cells (the upper right quadrant), and necrotic cells (the upper left quadrant). The percentage of cells in each quadrant was presented as the average of three independent experiments.

### 4.8. Protein Extraction and Western Blotting Analysis

After being plated in a 25 cm^2^ flask with 8 × 10^5^ cells, MDA-MB- 231 and T-47D were incubated at 37 °C with 5% CO_2_. The next day, cells were cultured for 48 h after being treated with SC at IC_50_ and ½ IC_50_ for both cell lines. In addition to a complete cocktail of protease inhibitors (# P8340, Sigma-Aldrich, St. Louis, MO, USA), cells were lysed using ice-cold lysis buffer (RIPA Lysis, Thermo Scientific, 25 mM Tris-HCl, pH 7.6, 150 mM NaCl, 1% NP-40, 1% sodium deoxycholate, and 0.1% SDS). The lysate was collected and then centrifuged at 4 °C and 12,000 rpm for 20 min. Protein concentrations of supernatants were determined using the BCA protein assay kit (Thermo Fisher Scientific Inc., Rockford, IL, USA). Protein samples were prepared by adding 2-mercaptoethanol (Fluka Sigma-Aldrich, St. Louis, MO, USA), distilled water, and 4X Laemmli buffer as a loading buffer, then denatured by heating at 75 °C for 10 min. The sample was separated on 10% SDS-PAGE, then transferred to nitrocellulose membranes, incubated in blocking buffer for 1 h at room temperature, then incubated overnight at 4 °C with primary antibodies P.AKT (abx121832, 1:500), P.ERK 1/2, ERK1/2 (#9101& #4695, 1:1000), P.mTOR (#YH4020740A, 1:2000), PTEN (#9188, 1:1000), PDCD4 (#9535, 1:1000), Pro-caspase 3 (#14220, 1:1000), Caspase 3 cleaved (#9664, 1:1000), and β-actin (#4970, 1:1000). The membranes were rinsed in Tris-buffered saline with Tween20 (TBST) the next day, then treated with the secondary antibody (AP-linked Antibody, #7054, 1:3000) for 2.5 h before being washed and subjected to NBT/BCIP solution to examine the protein bands [138]. The membranes were photographed, and ImageJ software (Version 1.52p software 64, NIH, USA) was used to quantify the band intensities.

### 4.9. RNA Extraction and Quantitative Real-Time PCR (qRT-PCR)

Cells were cultured in 6-well plates at a density of 2 × 10^5^ cells per well and cultured for 24 h until 80–90% confluence. Then, cells were treated with SC at concentrations of ½ IC_50_ and IC_50_ and incubated for 48 h. Total RNA, including miRNAs, was extracted from cultured cells using Qiazol reagent according to the manufacturer’s protocol for the miRNeasy Mini kit (No. 217004). The miScript II RT Kit (No. 218161) was then used to convert the miRNAs and other RNA species to cDNAs. Total RNA concentration and purity were measured using Thermo Scientific’s NanoDrop 1000 Spectrophotometer (Waltham, MA, USA) and expressed as µg/µL. The levels of miR-21-5p (Accession number: MIMAT0000076, mature hsa miR-21-5p sequence (UAGCUUAUCAGACUGAUGUUGA)) and miR-196a-5p (Accession number: MIMAT0000226, mature hsa miR-196a-5p sequence (UAGGUAGUUUCAUGUUGUUGGG)) were measured using Qiagen’s miScript Primer Assays for miR-21-5p and miR-196a-5p. Real-time PCR was carried out with the miScript SYBR Green PCR Kit (No. 218073). RNU6B was used as a reference for miRNA expression. The fold change in miR-21-5p and miR-196a-5p expression was estimated using the gene comparative approach (2^−△△^CT method). All measurements were carried out three times with the Bio-Rad CFX96 real-time PCR instrument. cDNA templates of other RNA species were used to measure the transcription of target genes (PTEN and PDCD4), long non-coding RNA GAS5 and HIF1-alpha, as well as apoptotic regulator genes (Bax, Bad, Bcl2, Caspase-8, Caspase-9, and Caspase-3) in treated cells. qRT-PCR was performed using Vivantis ViPrime PLUS Taq qPCR Green Master Mix I kit (code QLMM12) to determine the levels of mRNA (Enzymatic activation at 95 °C for 2 min, then 40 cycles of denaturation for 15 s at 95 °C followed by annealing and data collection for 60 s at 60 °C); primer sequences are listed in Table 3. The mRNA expression levels were calculated relative to GAPDH using the gene comparative method (2^−△△CT^ method) [139]. All measurements were performed three times using the Bio-Rad CFX96 real-time PCR system.

### 4.10. Immunofluorescence Staining for PTEN Intracellular Protein

MDA-MB-231 and T-47D cells were placed on coverslips in 6-well plates (1 × 105 cells). After 24 h of adherence, they were treated with SC at a dose of IC50 and incubated for 48 h. The immunocytochemistry protocol was performed as follows [149]. Cells on coverslips were gently washed with PBS and fixed with 4% paraformaldehyde, followed by permeabilization with 0.2% Triton X-100 (#TB0198, Bio Basic Inc., Markham, ON, Canada). Then they were incubated with blocking buffer, PBST (1% BSA and 0.2% Tween prepared in PBS). The coverslips were incubated with anti-PTEN antibody (1:125) in 1% BSA overnight at 4◦C. After washing thrice in PBST, the cells were incubated with a fluorescent-labelled (Alexa Fluor (R) 488) secondary antibody (1:1000) in 1% BSA for 1 h at room temperature. After washing the cells, cell nuclei were counterstained with Hoechst 33342 staining dye solution (1 µg/mL): excitation 405 nm, emission 410–461 nm. A mounting medium with an anti-fade agent was used to mount the coverslips on the glass slides. Cells were examined under an inverted confocal microscope, Leica DMi8 (Leica, Wetzlar, Germany), equipped with a 63× oil immersion objective, and images were collected.

### 4.11. MiR-21 Mimic Transfection

Cancer cells were transfected with miR-21 mimic to investigate the targeting effect of SC on miR-21 at the molecular level. MDA-MB-231 and T-47D cells were seeded in 6-well plates under normal growth conditions for a short time before transfection. Cells were transfected with miR-21 mimics (5 nM, #339173, Qiagen, Germantown, MD, USA) or a corresponding scrambled mimic negative control (#YM00479903, Qiagen, Germantown, MD, USA) using HiPerfect transfection reagent (#301704, Qiagen, Germantown, MD, USA), according to the manufacturer’s protocols. After 24 h post- transfection, cells were treated with SC at its IC_50_ concentration and incubated for 48 h under normal growth conditions. After incubation, the cells were harvested and employed for RT-PCR analysis to validate the mimic transfection. The TargetScan and microRNA.org databases were used to predict the potential targets of miR-21. Total RNA, including miRNA, was extracted and prepared for RT-PCR as described above to measure the expression levels of miR-21 and its endogenous target genes, PDCD4 and PTEN.

### 4.12. Molecular Docking Study of SC and Pre-miR-21

This analysis aimed to show the possible interaction between the precursor miR-21 and SC. The 3D crystal structure of wild-type pre-miR-21 apical stem-loop was downloaded from the Protein Data Bank, http://www.rcsb.org/pdb (accessed on 20 July 2026) (PDB ID: 5UZT), and used for this study. PDB ID: 5UZT represents a 31-nucleotide truncated pre-miR-21 apical stem–loop construct corresponding to residues G22–C52 of the pre-miR-21 primary sequence. It therefore includes the upper stem, the apical loop, and the local junctional region surrounding the reported 5′ Dicer-cleavage-site nucleotide A29. Initially, the crystallized water molecules were excluded from the downloaded crystal structure. Molecular preparation was done to add the hydrogen atoms. Energy minimization was performed by applying the MMFF94 force field. Then, the hydrogen atoms were hidden to clarify the interaction areas. The 2D structures of SC and streptomycin (as an inhibitor for miR-21) were downloaded from the PubChem database as an SDF file, optimized with ChemDraw Ultra 14.0 software, and saved in mol2 format. Protonation of the tested compound and the energy of the molecule were minimized by applying the MMFF94 force field. The docking process was done using the Ligand Scout 2.0 software using the blind docking technique. About twenty poses were predicted, and the best orientation was chosen; then the 3D and 2D binding modes were generated by Biovia Discovery Studio Visualizer 2021, and all data were collected. The docking process also included target site prediction for SC binding based on the target site reported in the literature. Kcal/Mol observed the binding affinity of the ligand as a unit for a negative score.

### 4.13. Statistical Analysis

The results were expressed as individual values and as means ± standard deviation (SD) of the mean. Data were analysed by one-way analysis of variance (ANOVA) and were performed using the Statistical Package for the Social Sciences (SPSS, Chicago, IL, USA) program, version 25, followed by Tukey’s post hoc test to compare significance between multiple comparisons. *p* < 0.05 *, ^#^, *p* < 0.01 **, ^##^, and *p* < 0.001 ***, ^###^ indicate significant differences.

## 5. Conclusions

SC exhibits potent anticancer activity against both hormone-responsive and triple-negative breast cancer cells by inhibiting proliferation, colony formation, and migration, and by inducing cell -cycle arrest and apoptosis. These effects are associated with downregulation of miR-21, activation of PTEN and PDCD4, and inhibition of AKT/mTOR and ERK1/2 signaling. The findings suggest that SC may inhibit miR-21 through interconnected regulatory mechanisms: (1) transcriptional downregulation via HIF-1α suppression, (2) post-transcriptional sponging by GAS5 upregulation, and (3) potential interruption of miR-21 maturation by docking to pre-miR-21 at the Dicer cleavage site and/or terminal apical loop. By concurrently suppressing the oncogenic activity of miR-21, restoring expression of the tumor-suppressive lncRNA GAS5, and inhibiting the AKT/mTOR and ERK1/2 pro-survival signaling pathways, SC produces complementary and multi-level antitumor effects in breast cancer cells. These findings highlight SC as a multi-target structural scaffold with preliminary anti-proliferative activity. They justify further medicinal chemistry optimization to enhance its potency, alongside targeted delivery strategies, before in vivo efficacy and pharmacokinetic evaluation.

## Figures and Tables

**Figure 1:**
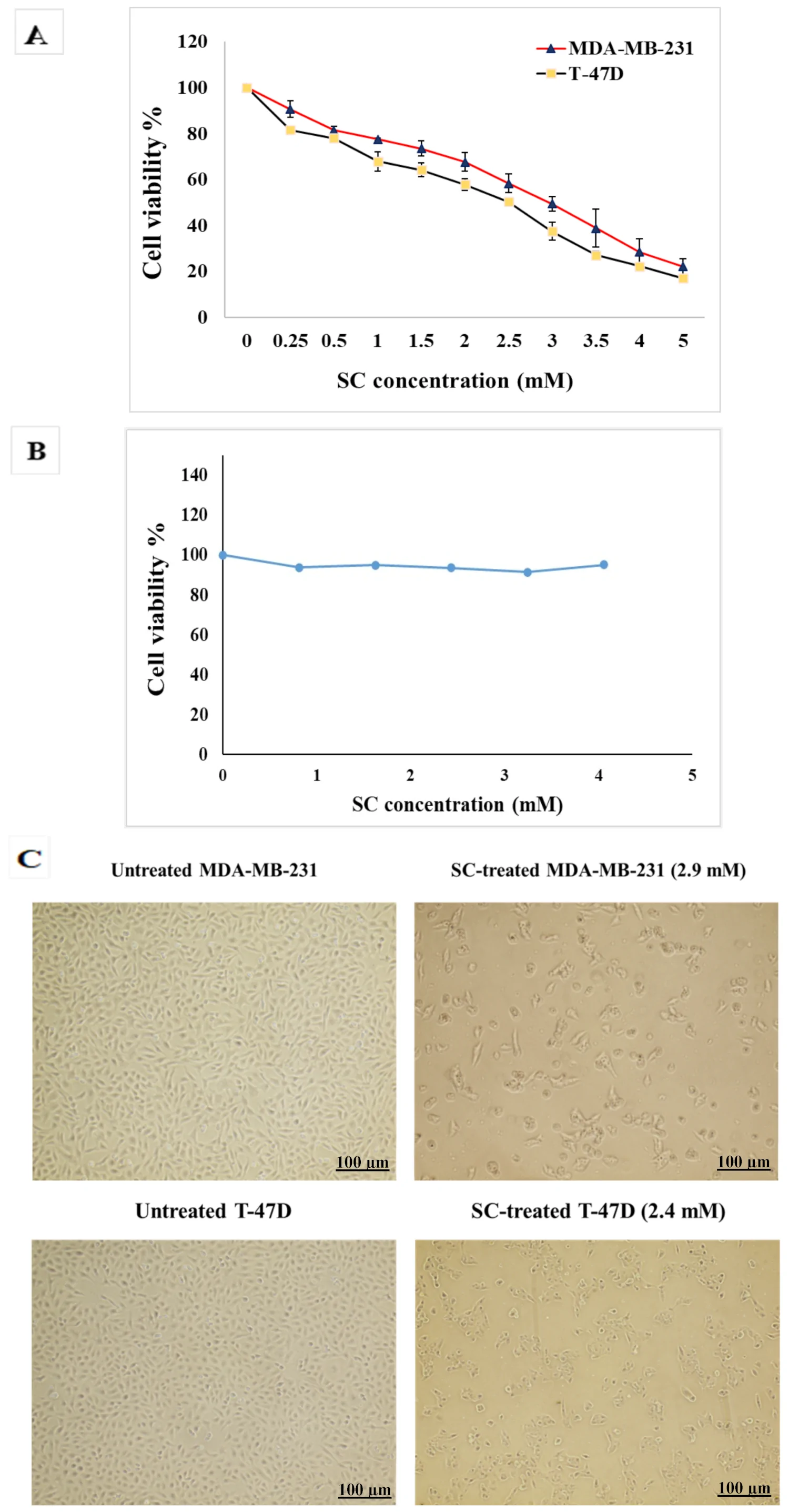

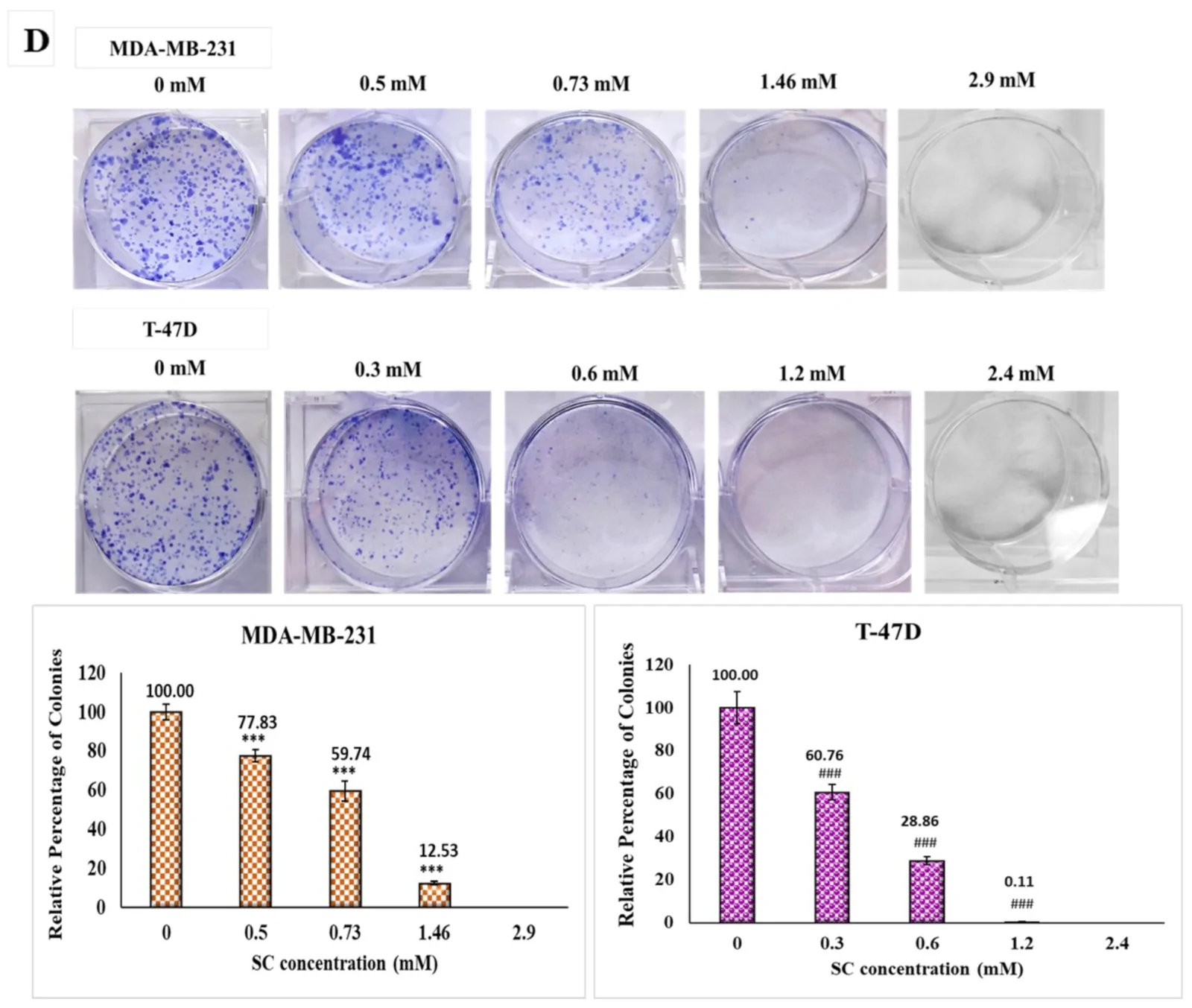

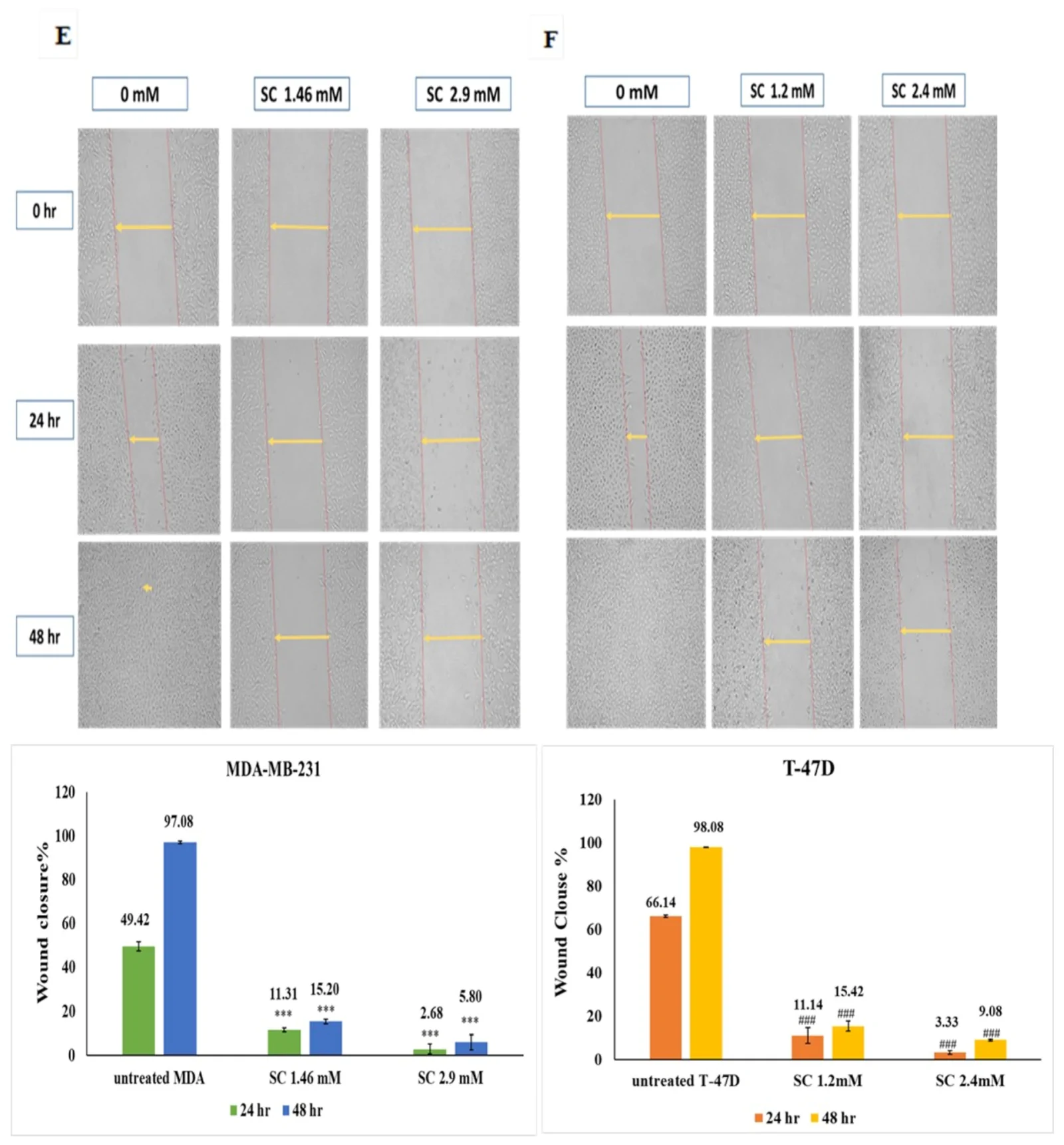

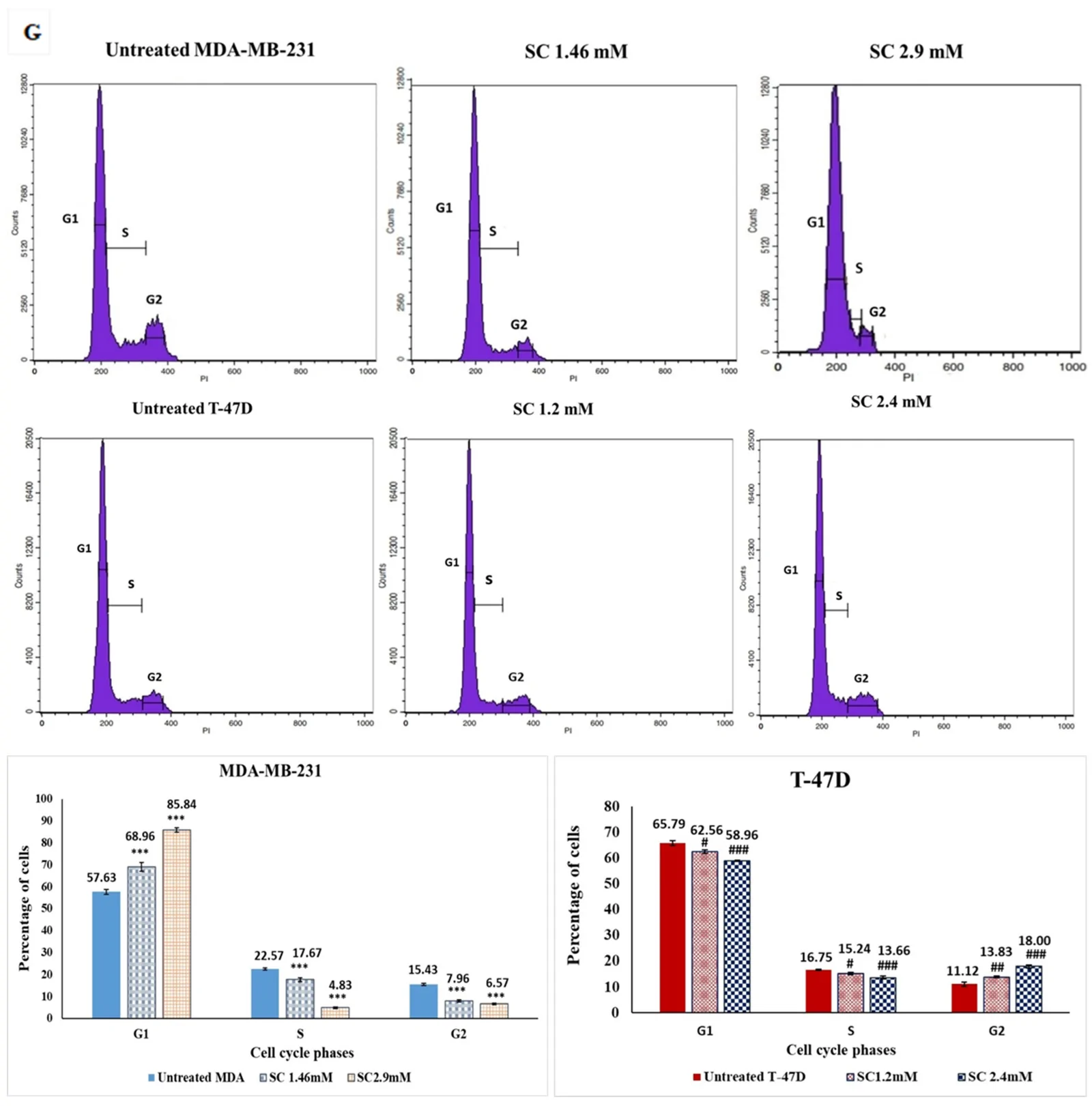

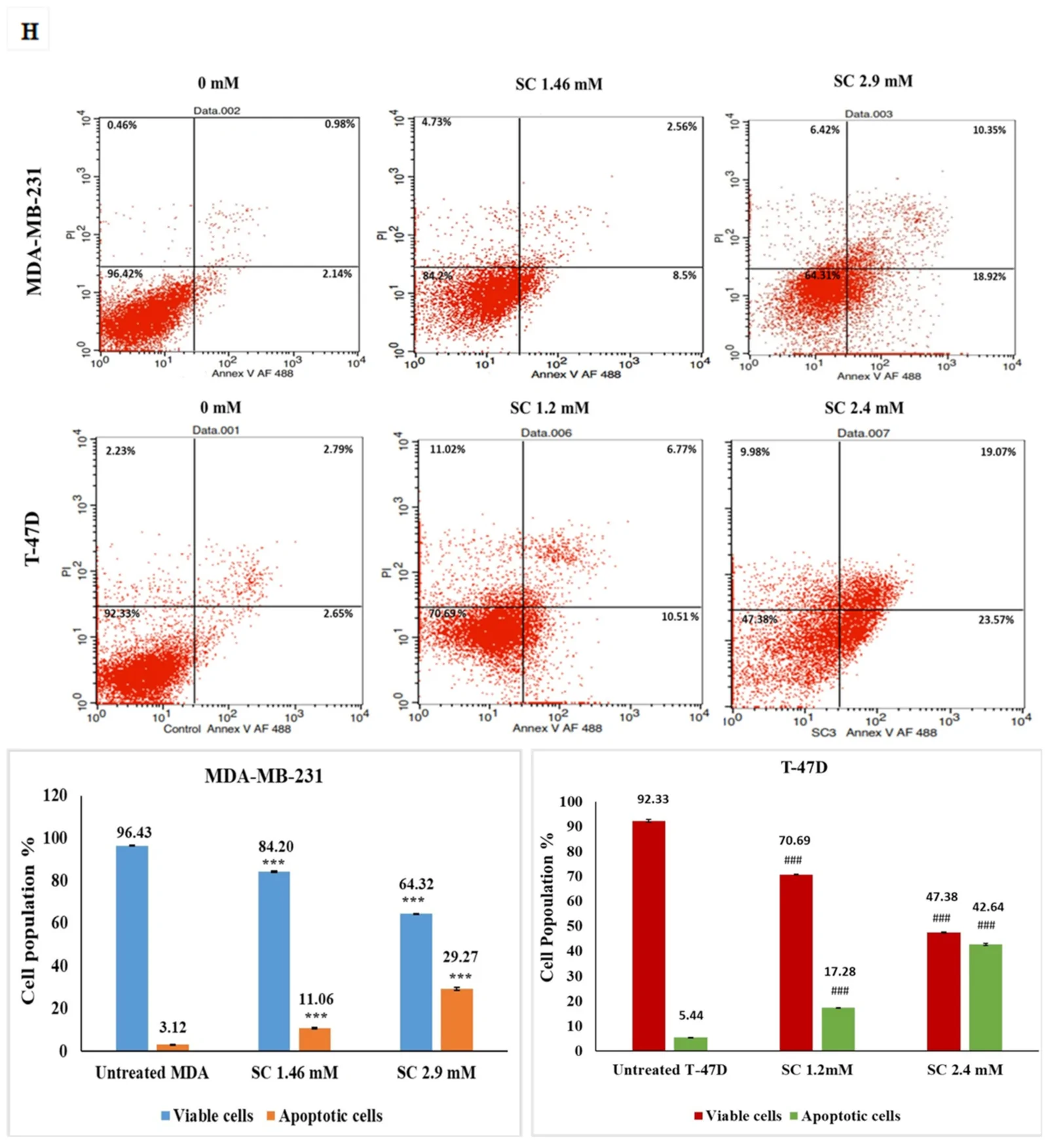
The anticancer effects of Sophocarpine against breast cancer cells. (**A**) Dose–response curve for cell viability in MDA-MB-231 and T-47D cell lines exposed to SC doses for 48 h. (**B**) Cytotoxicity of SC against a normal corneal fibroblast cell line after treatment with different doses of SC for 48 h. (**C**) Representative light microscopy images of SC-treated MDA-MB-231 and T-47D cells after 48 h at both IC_50_ doses. (**D**) SC inhibited colony-forming ability in MDA-MB-231 and T-47D cells at different concentrations for 48 h. The bar charts represent the mean percentage of colonies in SC-treated cells at different concentrations using the one-way ANOVA test. SC suppressed the migration ability of MDA-MB-231 (**E**) and T-47D cells (**F**) at IC_50_ and ½ IC_50_ after 24 and 48 h of exposure (Yellow arrows indicate the width of the wound gap, scale bar: 100 µm). (**G**) SC induced cell cycle arrest in the treated cell lines. Following a 48 h treatment with SC at ½ IC_50_ and IC_50_, MDA-MB-231 and T-47D breast cancer cells were harvested for cell cycle analysis by flow cytometry. The bar chart shows the proportion of MDA-MB-231 and T-47D cells in each phase of the cell cycle. (**H**) SC induced a proapoptotic effect in the treated cell lines. Following 48 h treatment with SC at ½ IC_50_ and IC_50_, MDA-MB-231 and T-47D cells were analyzed by flow cytometry for apoptosis. Early apoptosis (AV+/PI−) appears in the lower-right panels, and late apoptosis (AV+/PI+) in the upper-right panels. Bar charts show percentages of apoptotic and viable cells in the treated cell lines. The results are shown as mean ± SD of three independent experiments, and statistical analysis was performed using one-way ANOVA with Tukey’s post hoc test (^#^ *p* ≤ 0.05, ^##^ *p* ≤ 0.01, ***^, ###^ *p* ≤ 0.001) versus untreated cells.

**Figure 2:**
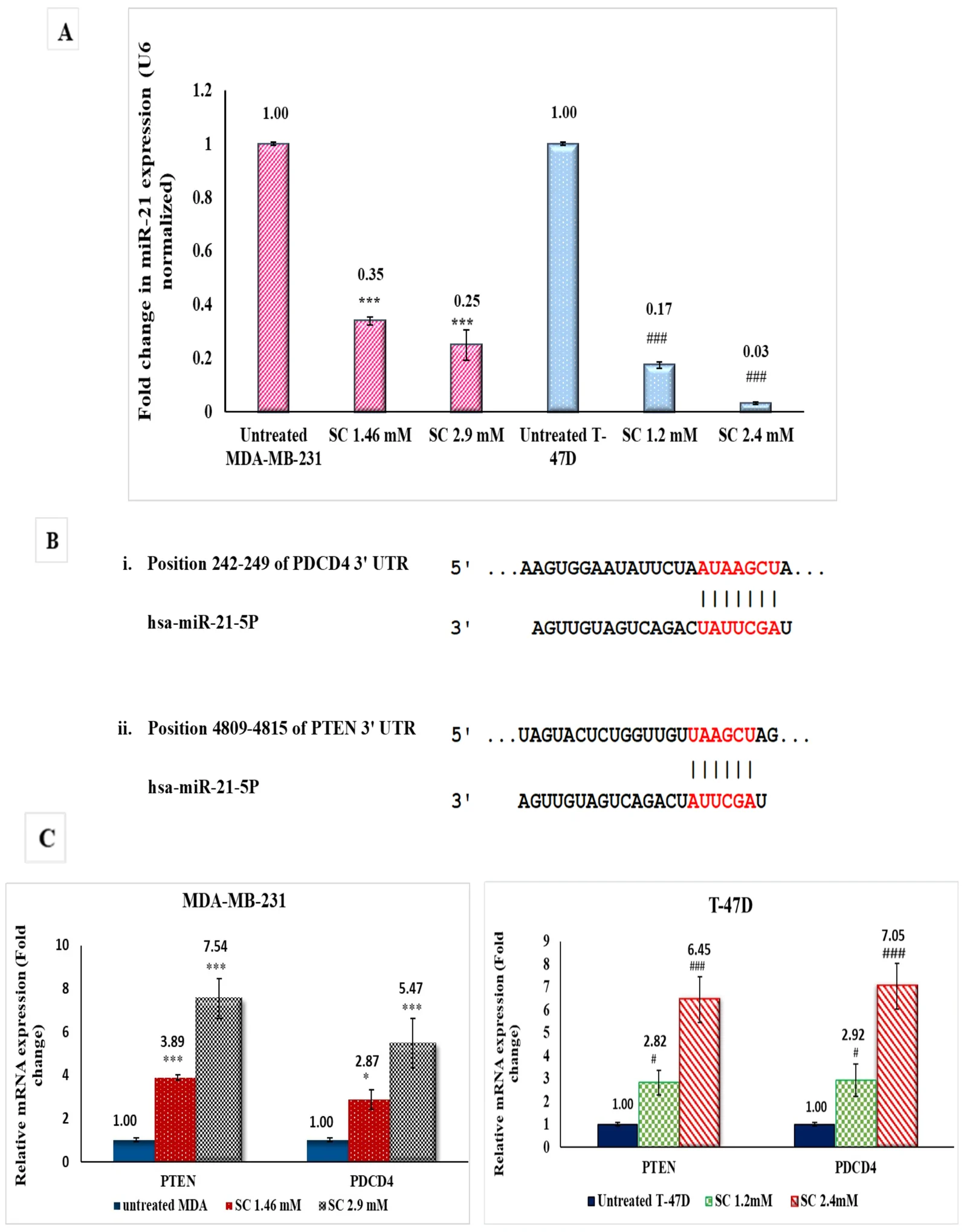

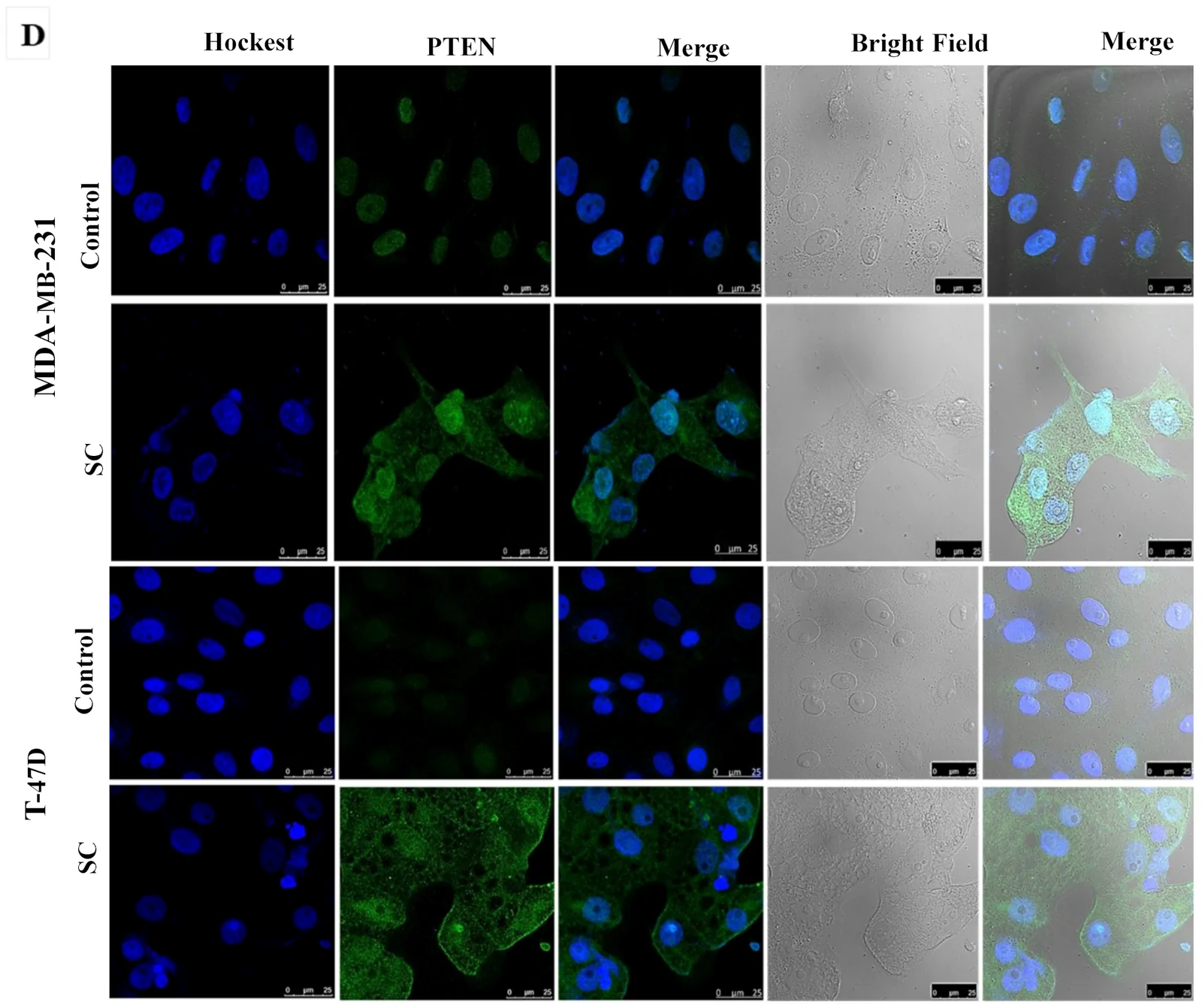

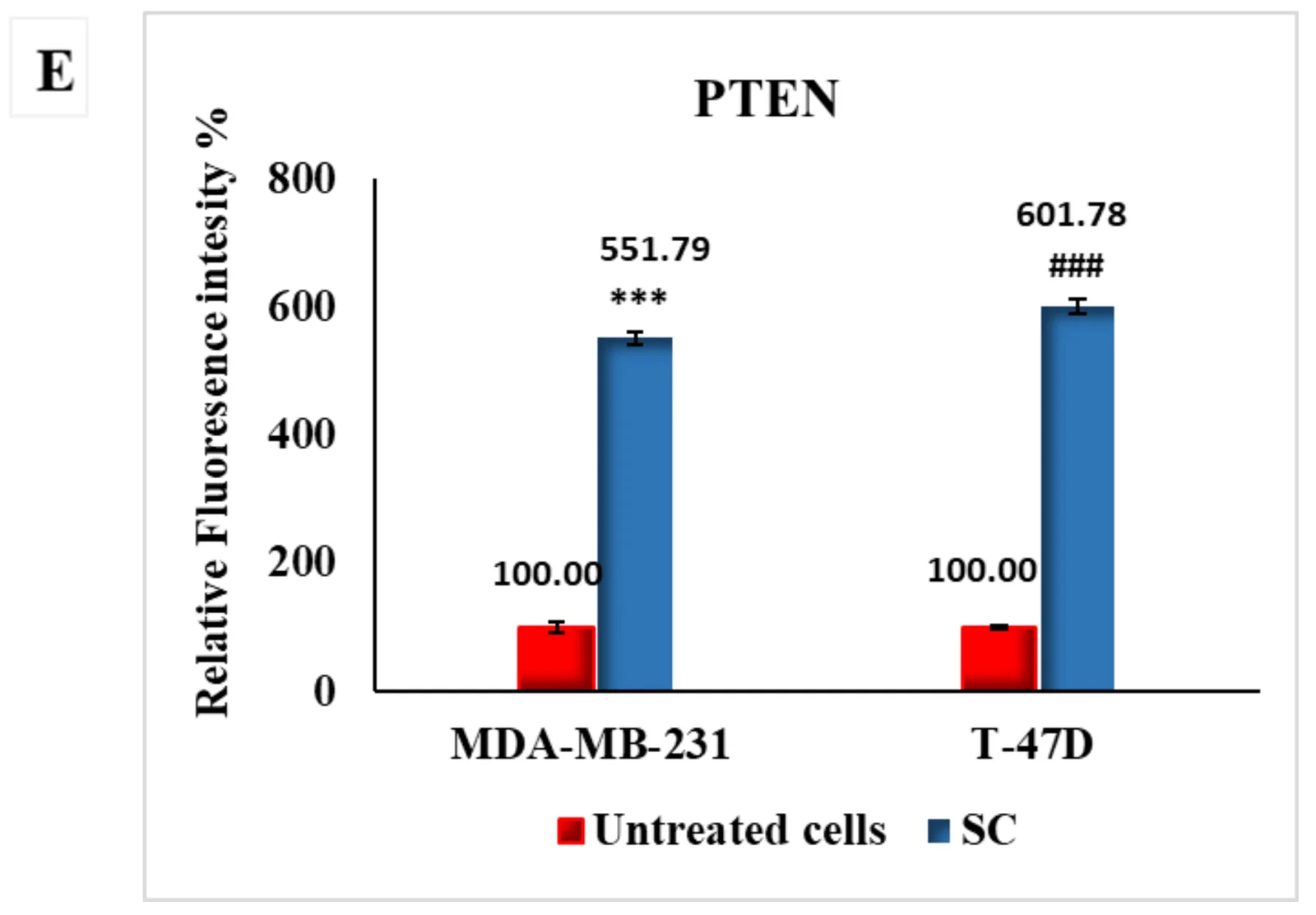
SC inhibited miR-21 levels and upregulated its target genes in breast cancer cells. miR-21 expression was determined by qRT-PCR after SC treatment at ½ IC_50_ and IC_50_ for 48 h and normalized using RNU6B as a reference gene (**A**). miR-21 binding sites in the 3′ UTR of the human PDCD4 and PTEN transcripts (**B**). SC upregulated the expression of PTEN and PDCD4 at ½ IC_50_ and IC_50_ for 48 h using qRT-PCR and normalized using GAPDH as a reference gene, compared to untreated cells (set to 1.00) (**C**). Bar graphs represent the average fold change as Mean ± SD of three separate experiments. Breast cancer cell lines were treated with SC at the IC_50_ dose for 48 h, stained for PTEN (green), and counterstained with Hoechst 33342 staining dye (blue) to visualize the nuclei. Two cell lines were stained in triplicate, and typical pictures are displayed, the scale bar in the bottom right corner represents 25 µm (0–25 µm) (**D**). The bar graph represents the relative fluorescence intensity of intracellular PTEN in breast cancer cell lines, as the mean ± SD of three separate experiments (**E**). Statistical analysis was performed using one-way ANOVA with Tukey’s post hoc test; *p* values: *, ^#^ < 0.05, ***, ^###^ < 0.001. (*) compared to MDA-MB-231 untreated cells, (#) compared to T-47D untreated cells.

**Figure 3:**
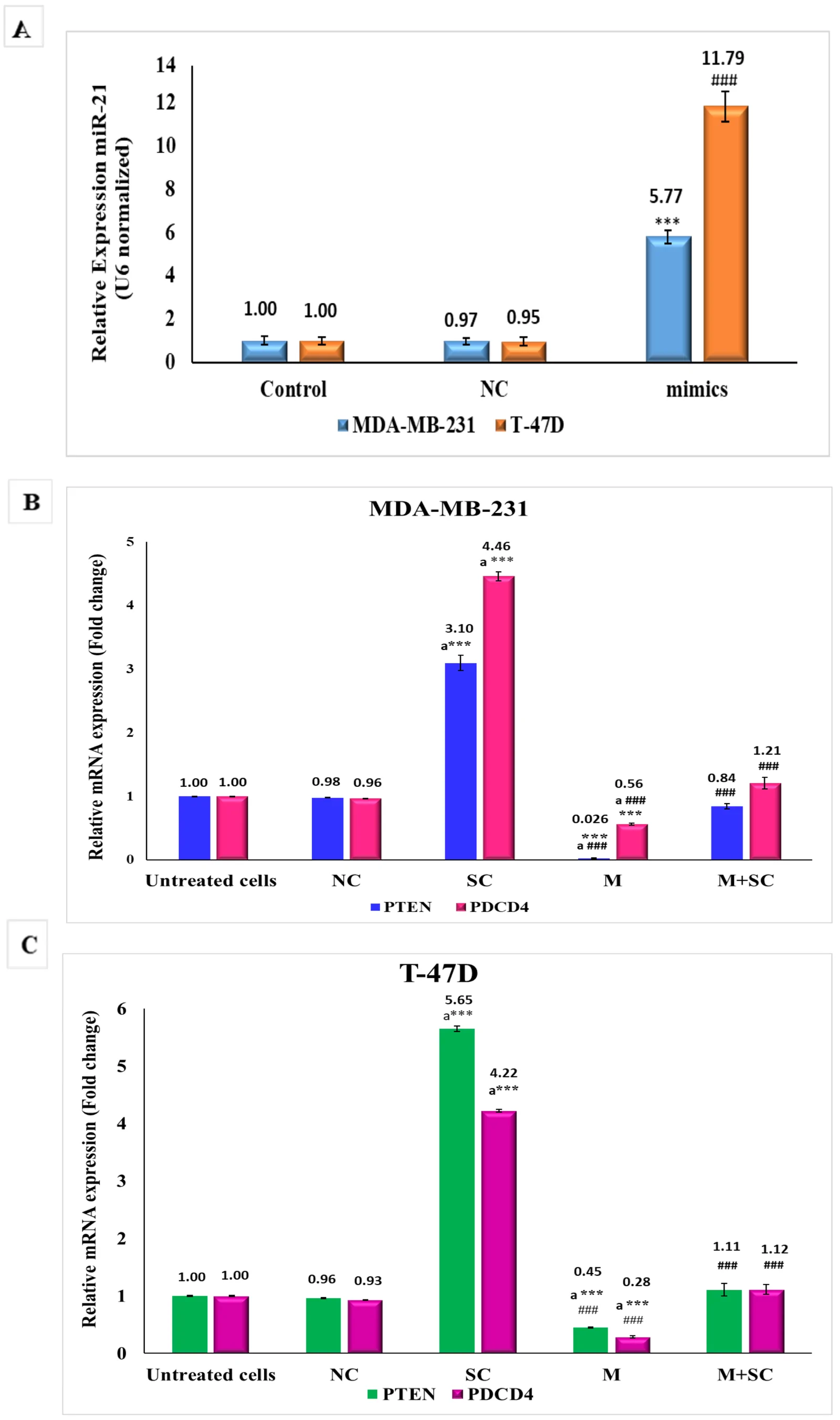
Effect of SC on PTEN and PDCD4 transcription after transient enhanced expression of miR-21. qRT-PCR analysis was used to evaluate the relative expression (fold change) of miR-21 after hsa-miR-21 mimic transfection at a concentration of 5 nM using HiPerfect transfection reagent for 48 h in both cell lines (**A**). PDCD4 and PTEN expression levels were determined by qRT-PCR after SC treatment at the IC_50_ dose for 48 h alone or with mimic transfection and normalized using GAPDH as a reference gene in MDA-MB-231 cells (**B**) and T-47D cells (**C**). *p* value: ***, ^###^ < 0.001. (*) Significant against untreated cells (set to 1.00); (a) significant (*p* < 0.001) against negative control (scrambled mimics) (NC); (#) significant against SC group. (M) Mimic transfection; (M+SC) SC treatment after mimic transfection.

**Figure 4:**
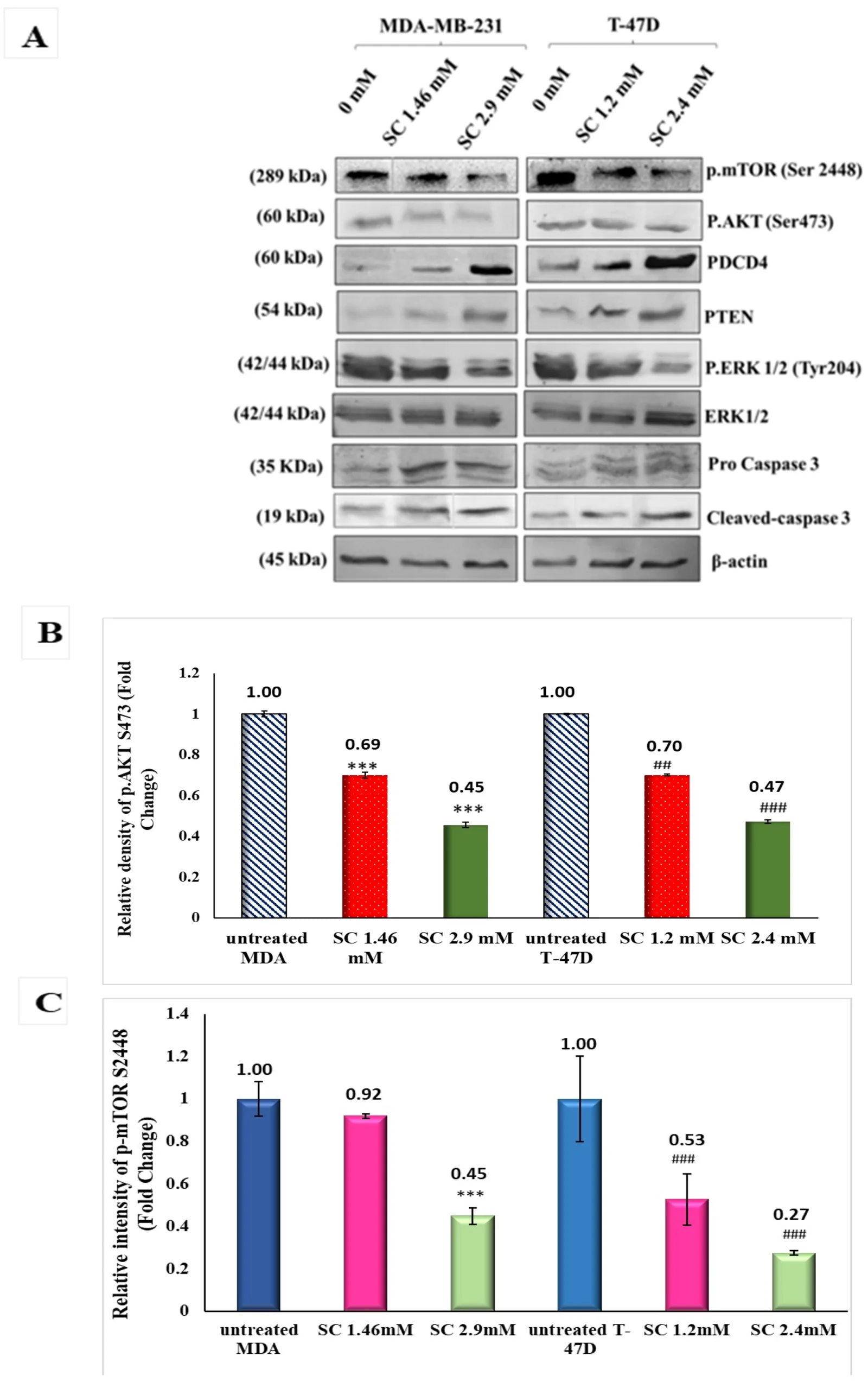

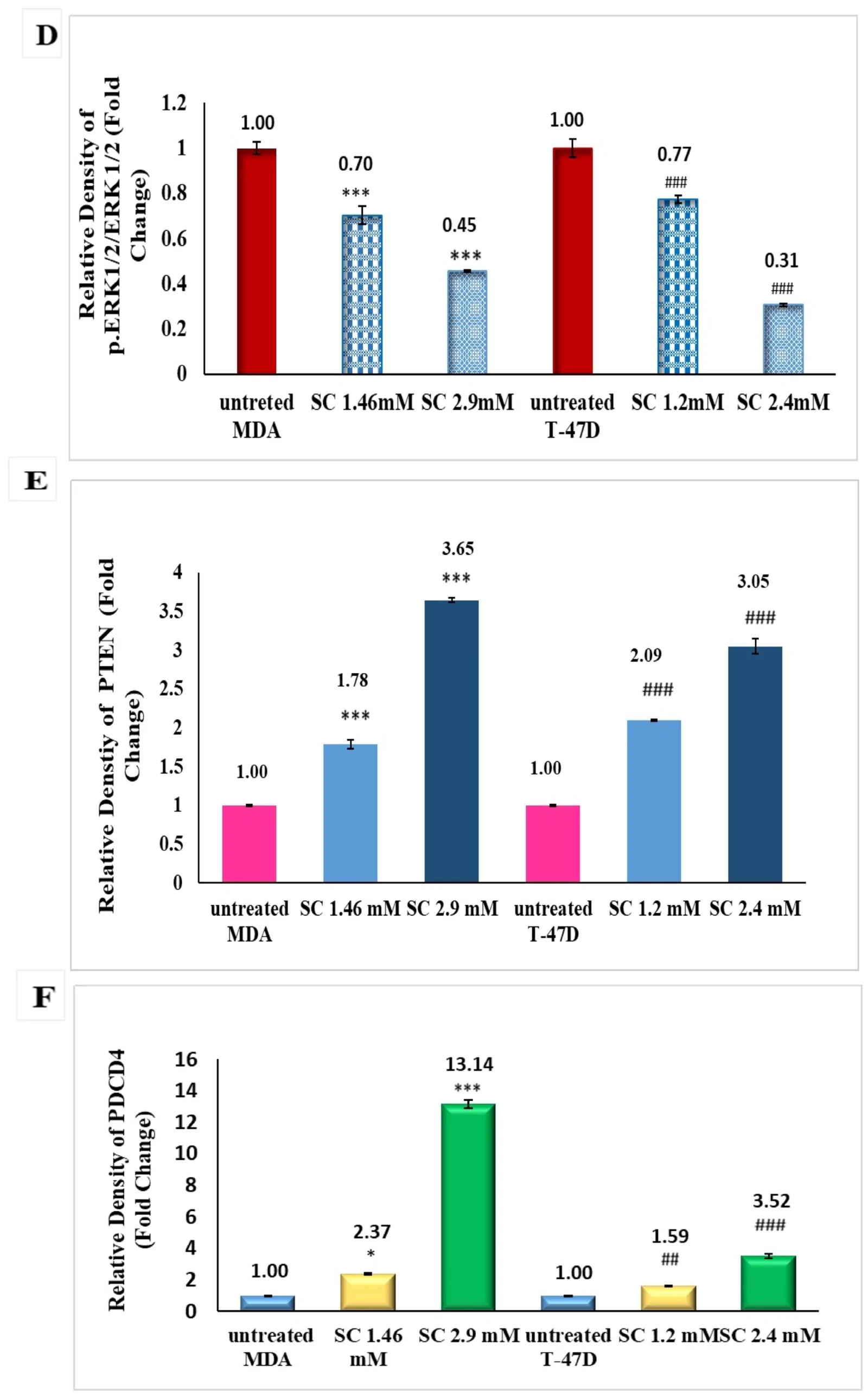

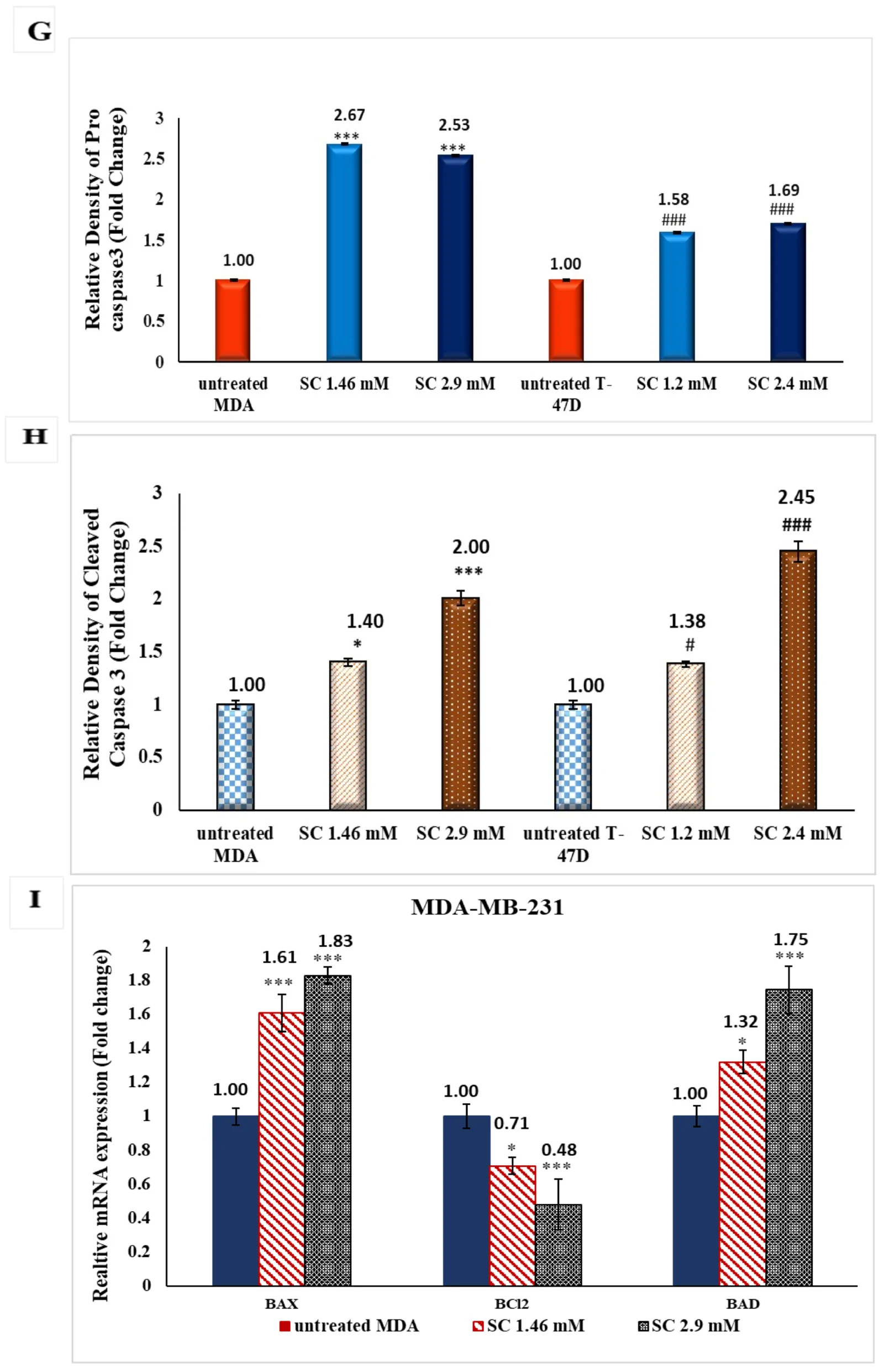

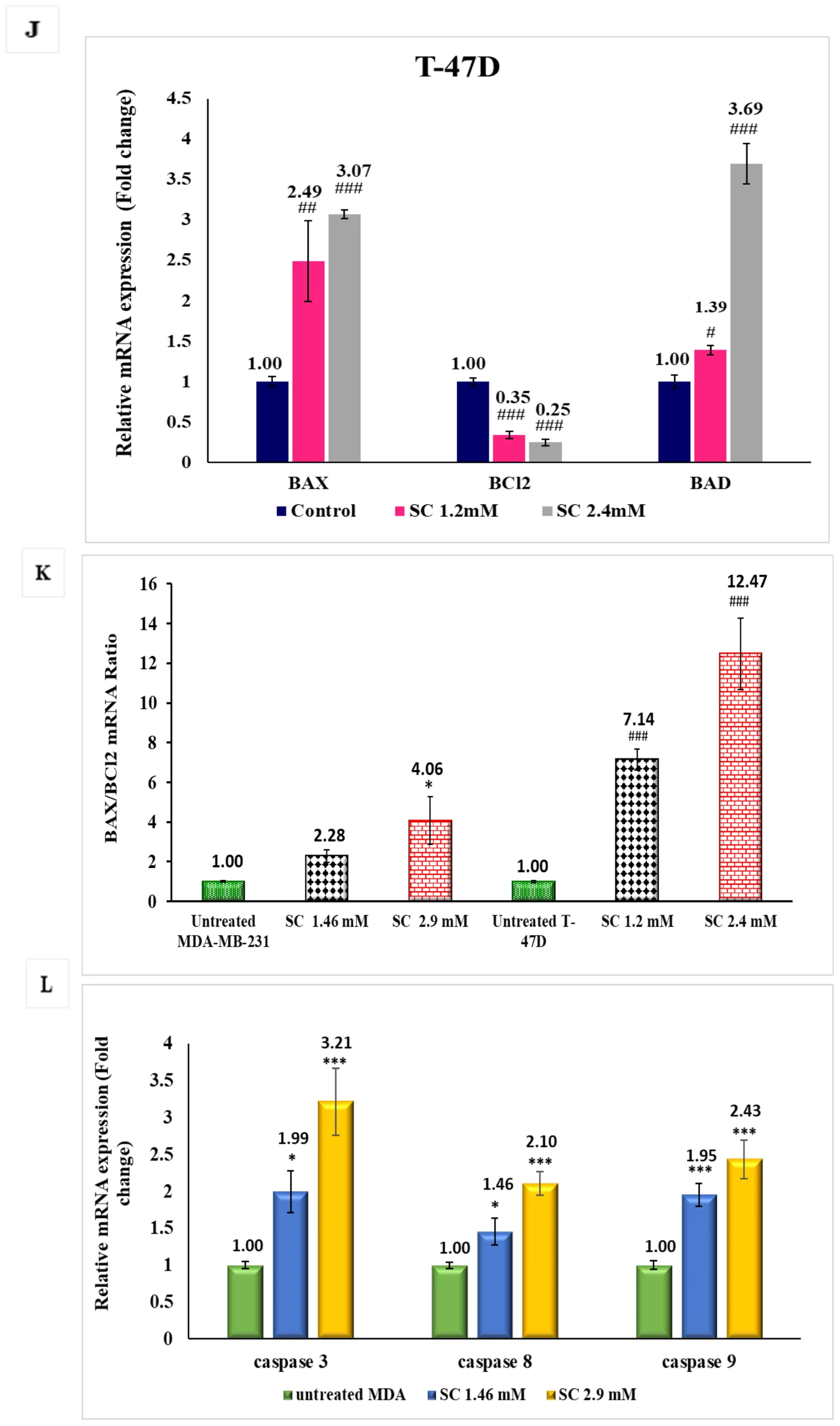

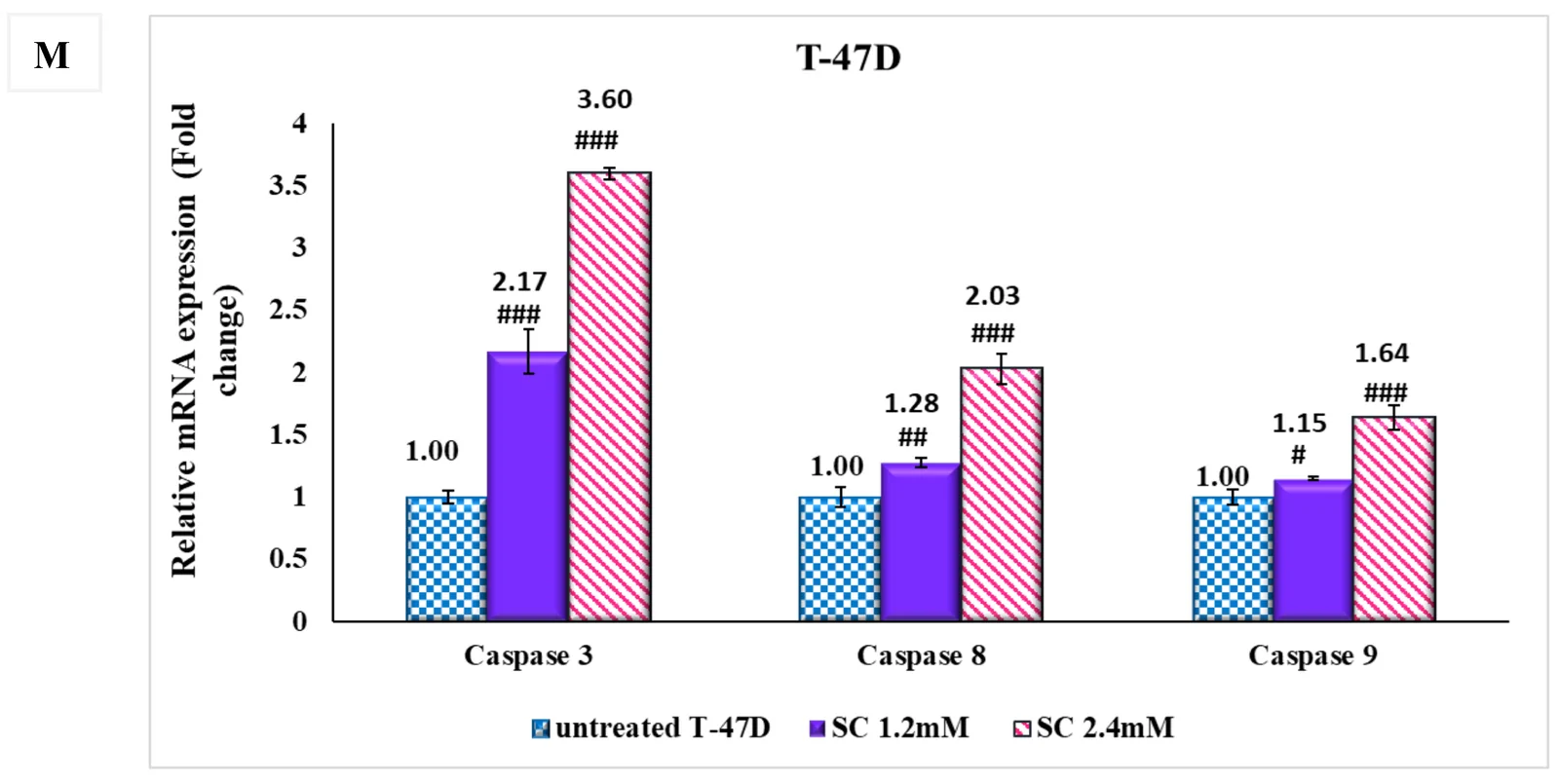
SC inhibits AKT/mTOR and ERK1/2 signaling pathways and promotes apoptosis in breast cancer cell lines. Western blot analysis of signaling parameters P.AKT (S473), P.mTOR (S2448), PDCD4, PTEN, ERK1/2, P.ERK1/2 (Thr202/Tyr204), pro-caspase 3, and cleaved caspase3 after SC treatment at ½ IC_50_ and IC_50_ for 48 h; β-actin was used as the loading control (**A**). Bar graphs represent the relative density of P.AKT (**B**), P.mTOR (**C**), P.ERK1/2/ERK1/2 (**D**), PTEN (**E**), PDCD4 (**F**), Pro-caspase3 (**G**), and cleaved caspase3 (**H**) in breast cancer cell lines, as the mean ± SD of three separate experiments. The band density was quantified using an image-analysing system (ImageJ software, Version 1.52p software 64, NIH, USA) by normalizing to β-actin. The shown blot is a representative image from three independent experiments. The dividing lines indicate that these bands were rearranged. SC modulates apoptotic gene expression in breast cancer cell lines. SC upregulated BAX and BAD transcription and downregulated BCl2 levels in MDA-MB-231 cells (**I**) and T-47D cells (**J**). BAX, BAD, and BCl2 mRNA expression levels were quantified by qRT-PCR after SC treatment at ½ IC_50_ and IC_50_ for 48 h, normalized to GAPDH as a reference gene and compared to untreated cells (set to 1.00). (**K**) BAX/BCl2 mRNA ratio in SC-treated MDA-MB-231 and T-47D cells. SC treatment enhanced transcription of caspase-3, caspase-8, and caspase-9 in MDA-MB-231 (**L**) and T-47D (**M**) breast cancer cells, as determined by qRT-PCR and adjusted for GAPDH. One-way ANOVA with Tukey’s post hoc test was used for statistical analysis; *p* values: *^, #^ < 0.05, ^##^ < 0.01, ***^, ###^ < 0.001. (*) compared to MDA-MB-231 untreated cells, (#) compared to T-47D untreated cells.

**Figure 5:**
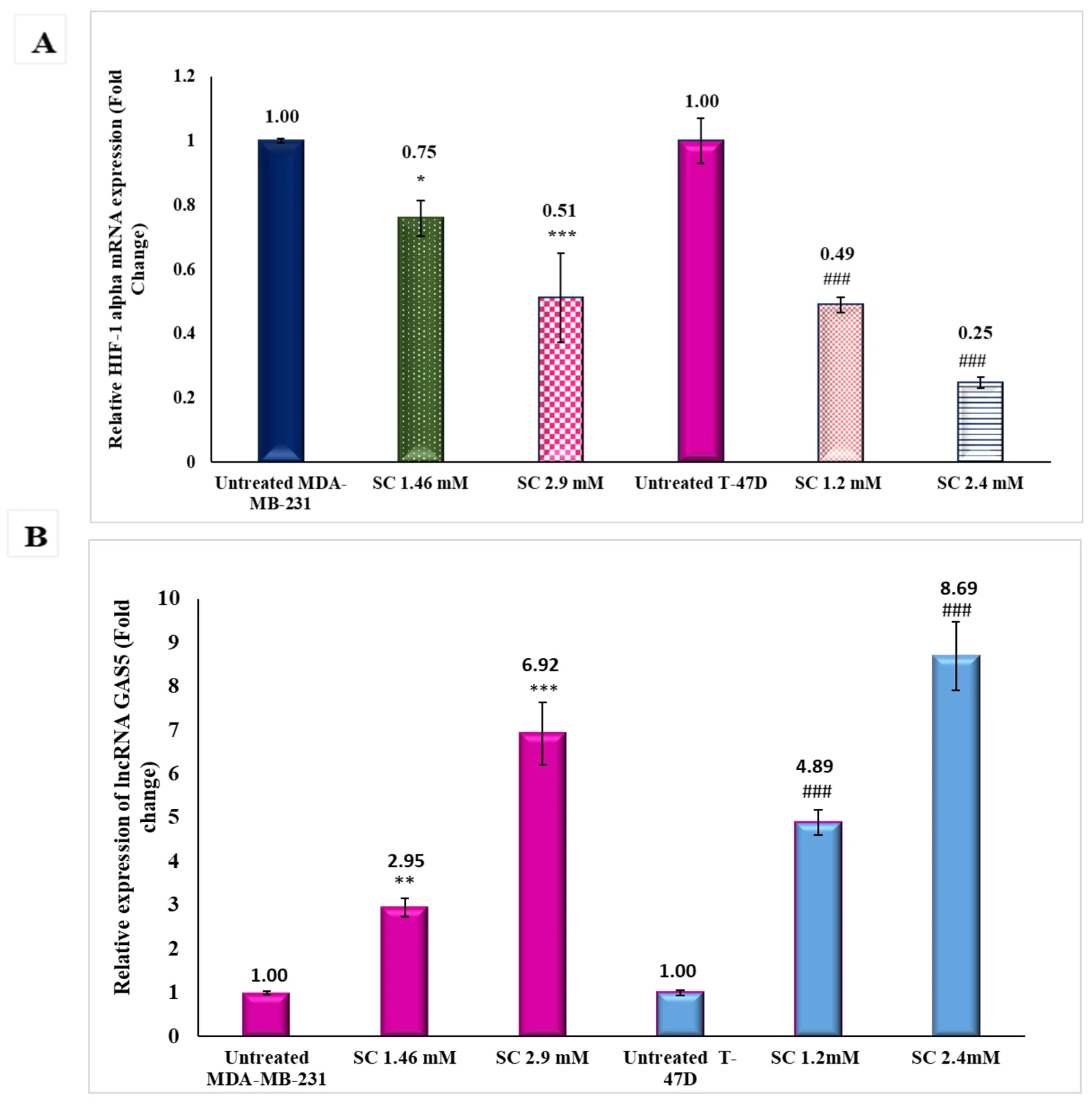

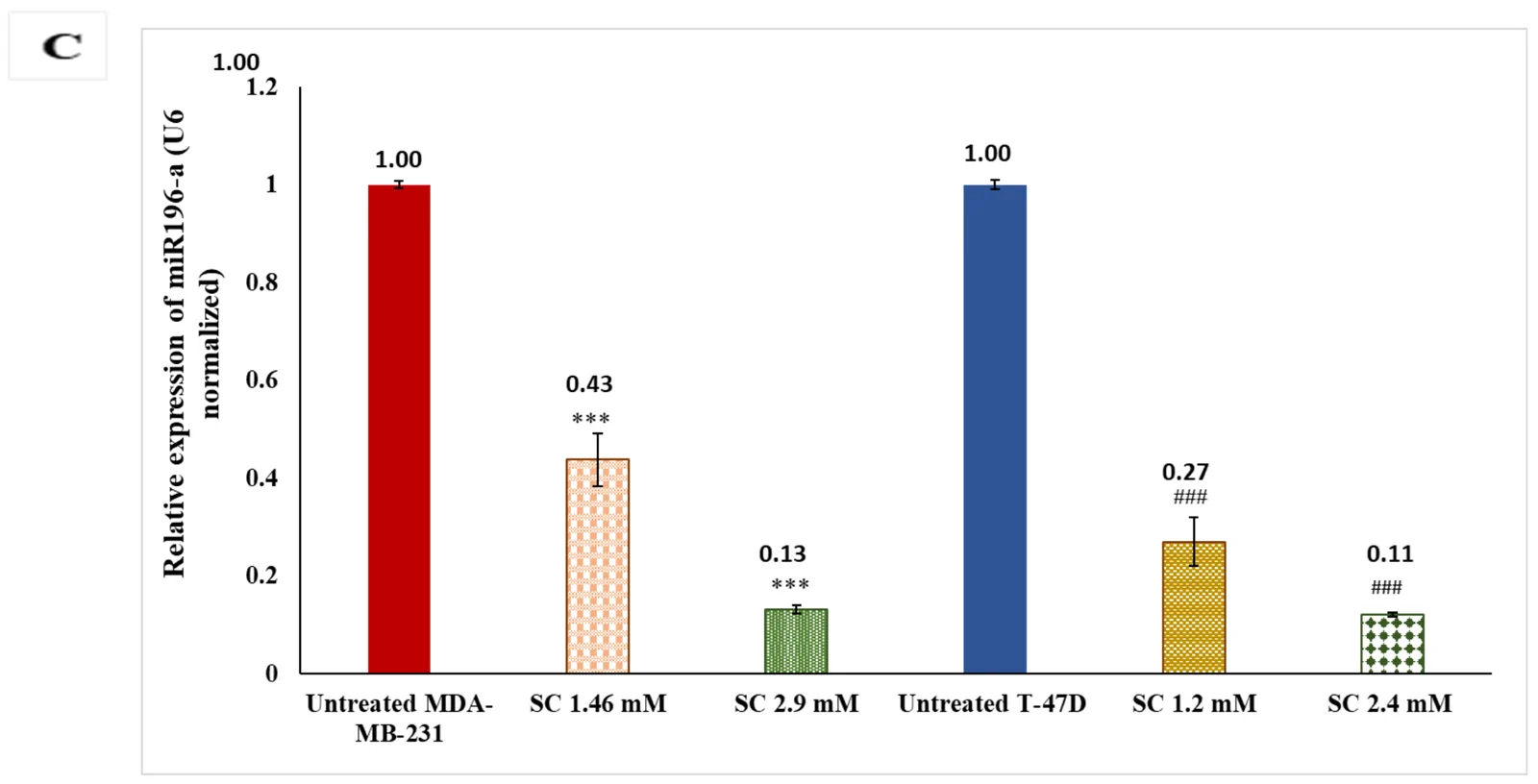
SC downregulated the transcriptional levels of HIF-1α and miR-196a-5p while enhancing the levels of lncRGAS5 in breast cancer cell lines. HIF-1α (**A**), lncRNA-GAS5 (**B**), and miR-196a-5p (**C**) transcripts were determined by qRT-PCR after SC treatment at concentrations of ½ IC_50_ and IC_50_ for 48 h and normalized using GAPDH and RNU6B as reference genes, compared to untreated cells (set to 1.00). Bar graphs represent the average fold change as Mean ± SD of three separate experiments. Statistical analysis was performed using one-way ANOVA with Tukey’s post hoc test, *p*-value: * < 0.05, ** < 0.01, ***^,###^ < 0.001. (*) compared to MDA-MB-231 untreated cells, (#) compared to T-47D untreated cells.

**Figure 6:**
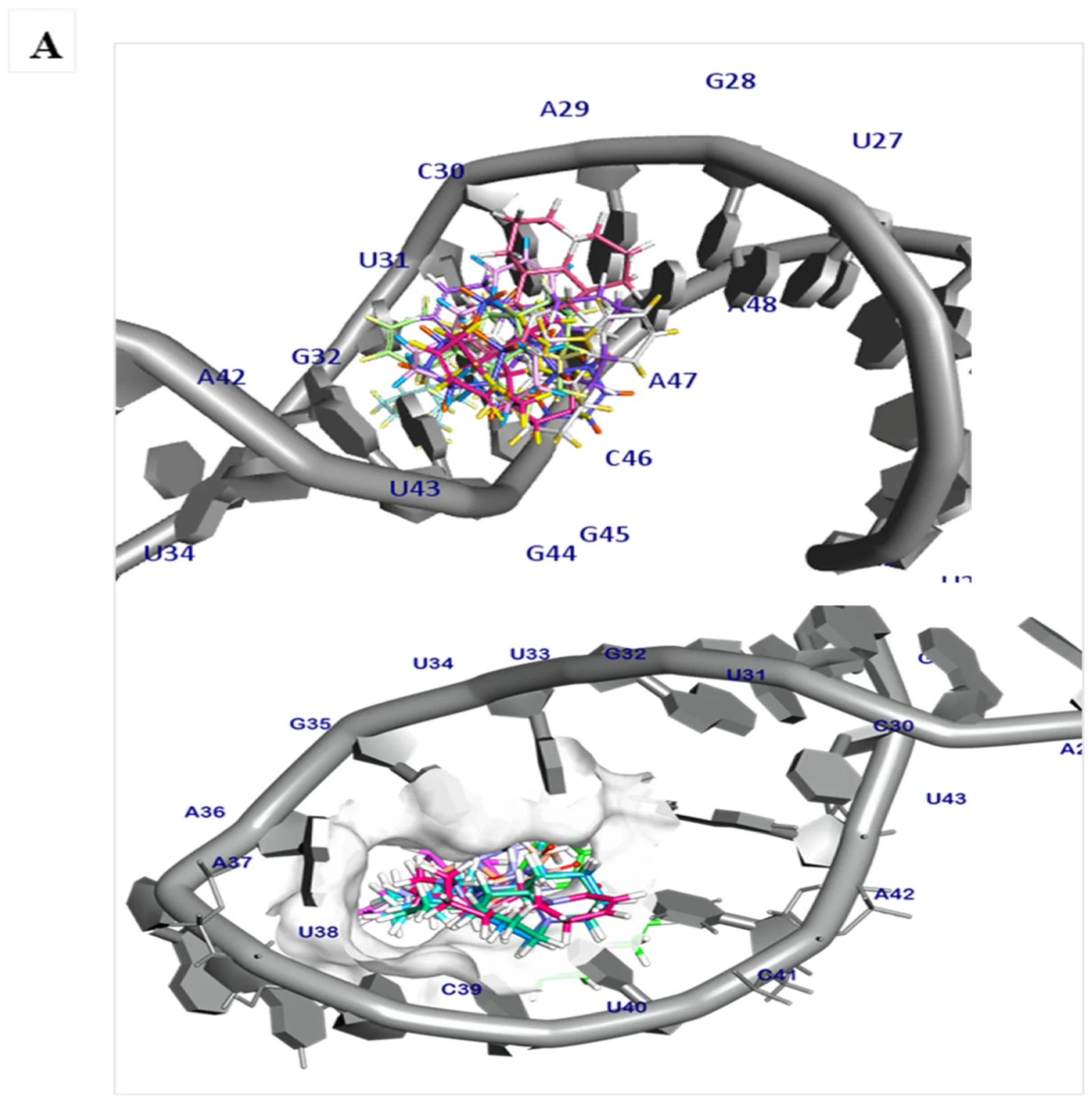

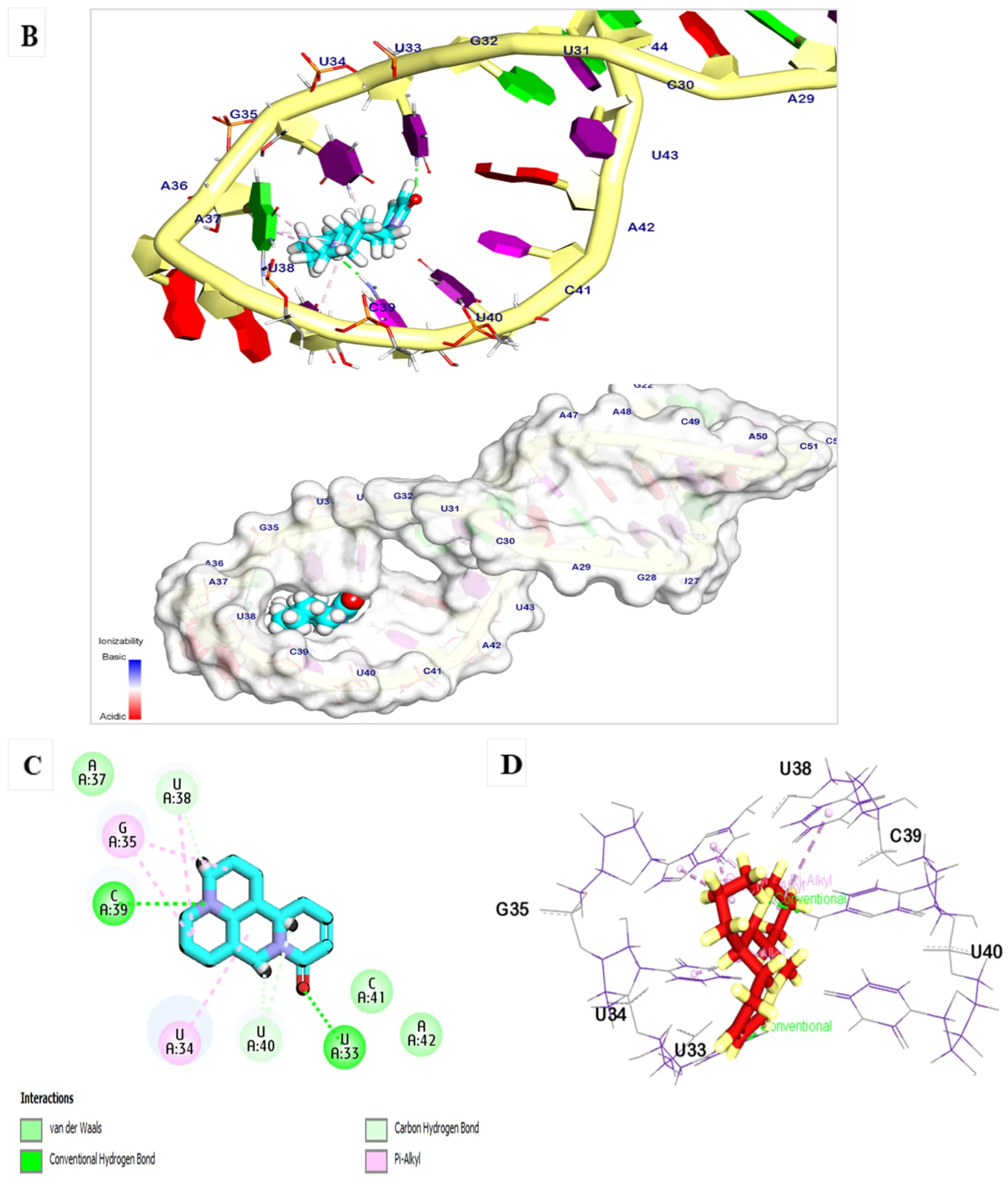

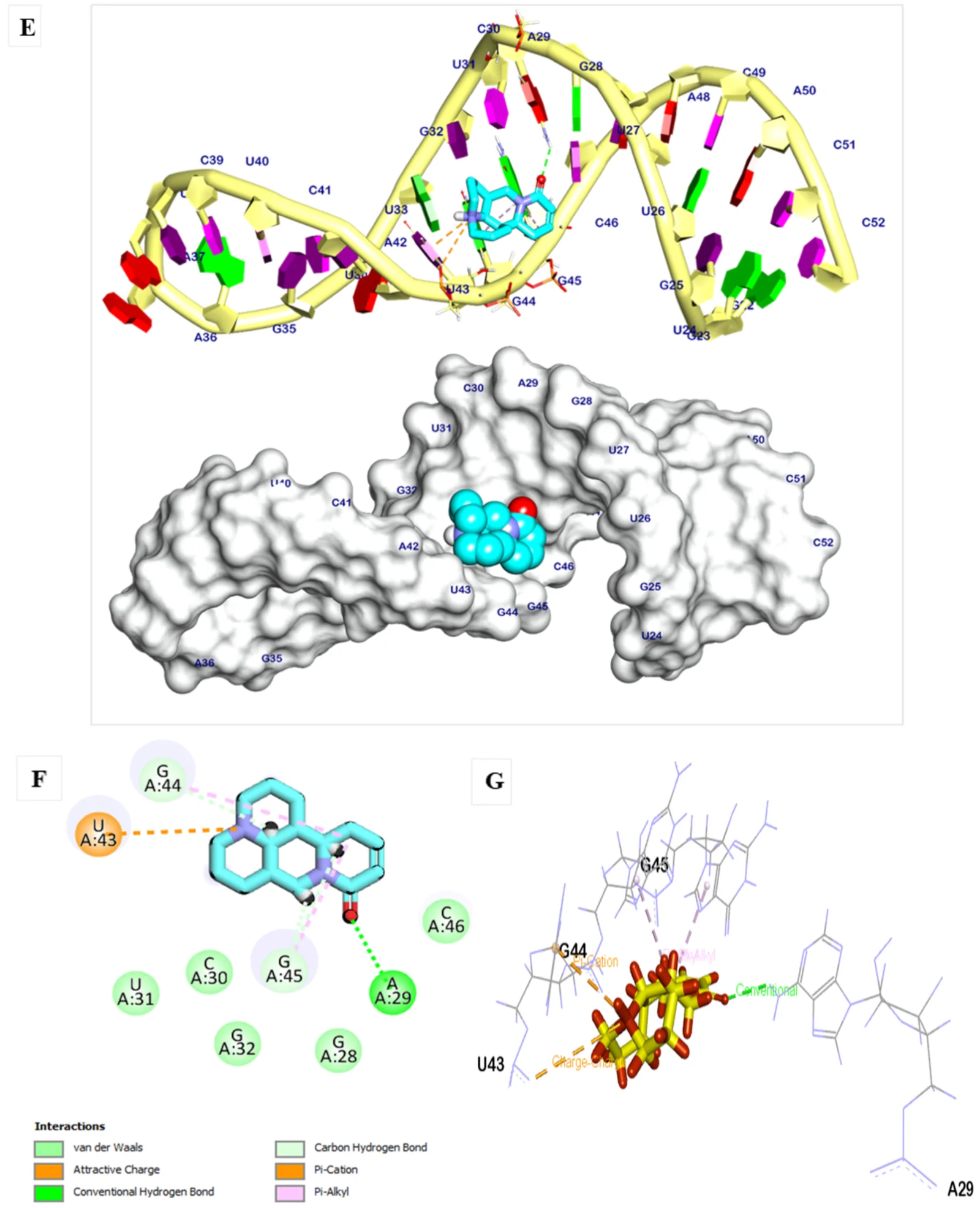

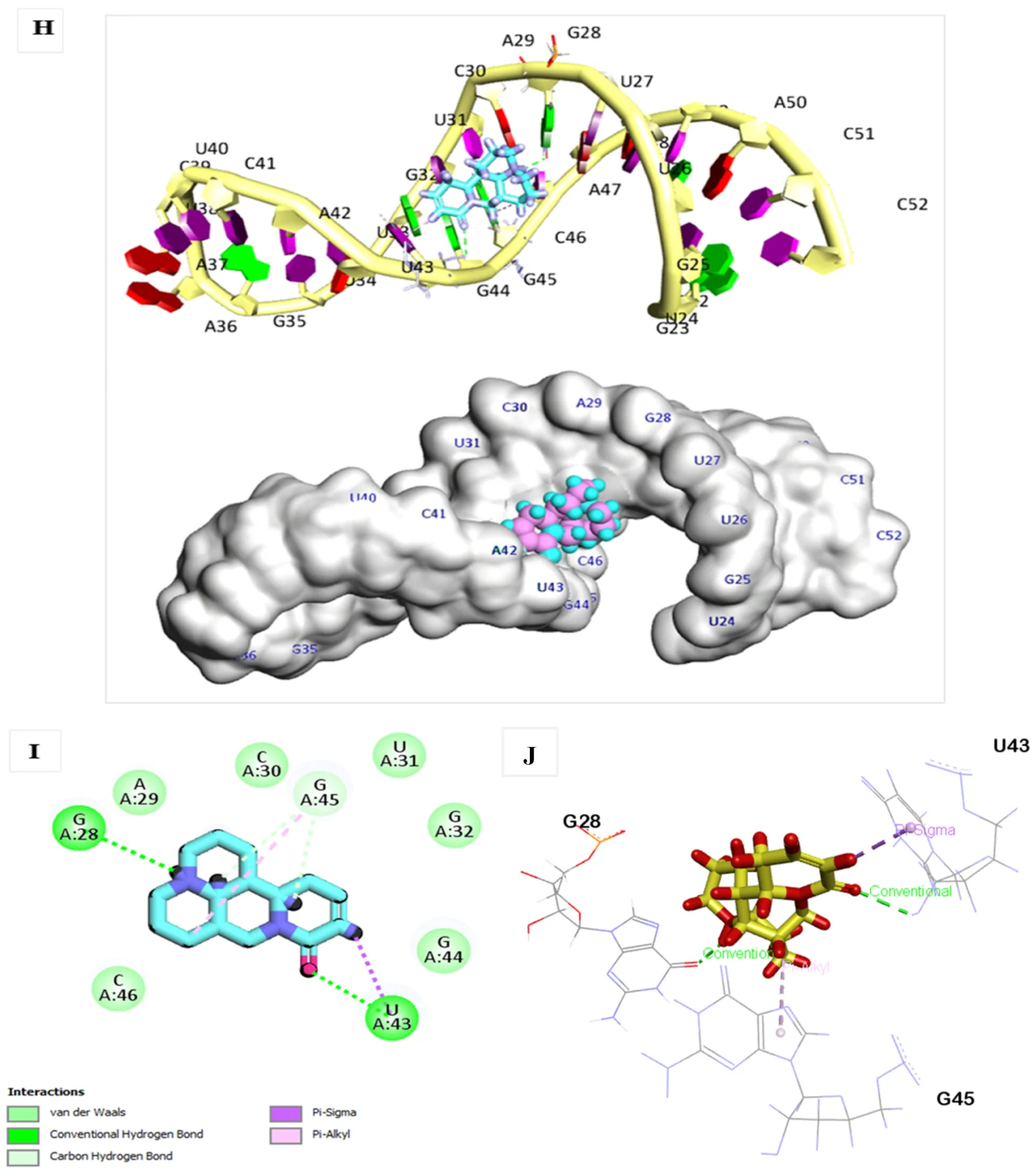
**Molecular docking of binding sites between SC and precursor miR-21**. SC binding modes (shown in different colors) against miR-21 precursor: in the terminal loop structure (lower) and at the stem-loop junction at the Dicer cleavage site of precursor miR-21 (upper) (**A**). 3D orientation and surface mapping of SC against the miR-21 precursor terminal loop (**B**). 2D structure of SC against miR-21 precursor terminal loop (**C**), SC docked in miR-21 precursor (terminal loop) (**D**), showing interacting nucleotides with two conventional hydrogen bonds (green color) and five pi-alkyl interactions (purple color). 3D orientation and surface mapping (**E**) and 2D structure of SC (**F**) against the miR-21 precursor stem-loop junction at the Dicer cleavage site. SC docked in the miR-21 precursor at the Dicer cleavage site (**G**), showing interacting nucleotides with conventional hydrogen bonds (green color), charged and pi-cation interactions (orange color), and two alkyl interactions (purple color). 3D orientation and surface mapping (**H**) and 2D structure of SC (**I**) against the miR-21 precursor stem-loop junction at the Dicer cleavage site (binding mode 4). SC docked in the miR-21 precursor stem-loop junction in binding mode 4 (**J**), showing interacting nucleotides with two conventional hydrogen bonds (green color), pi-alkyl interaction (purple color), and pi-sigma interactions (violet color).

**Figure 7:**
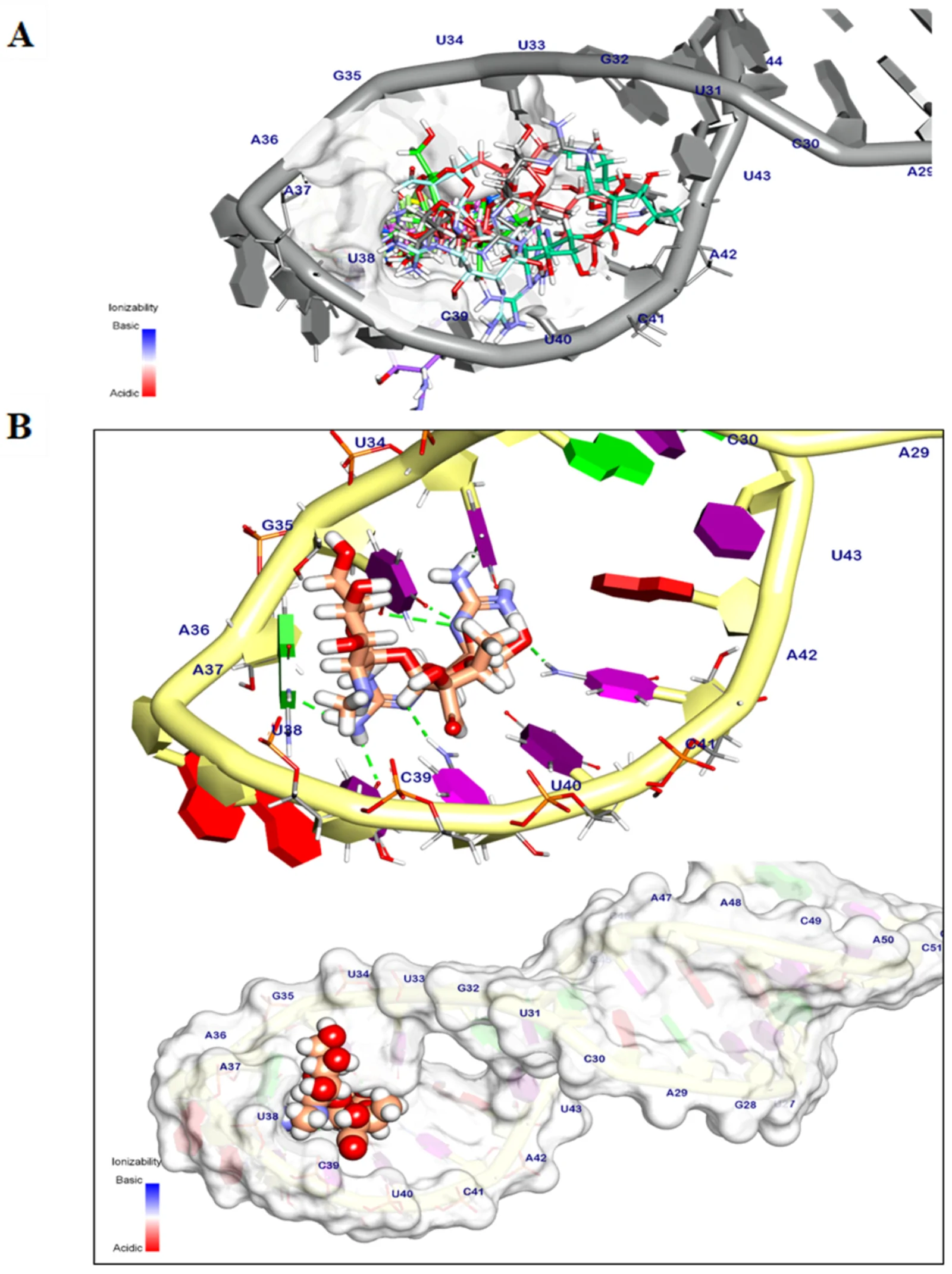

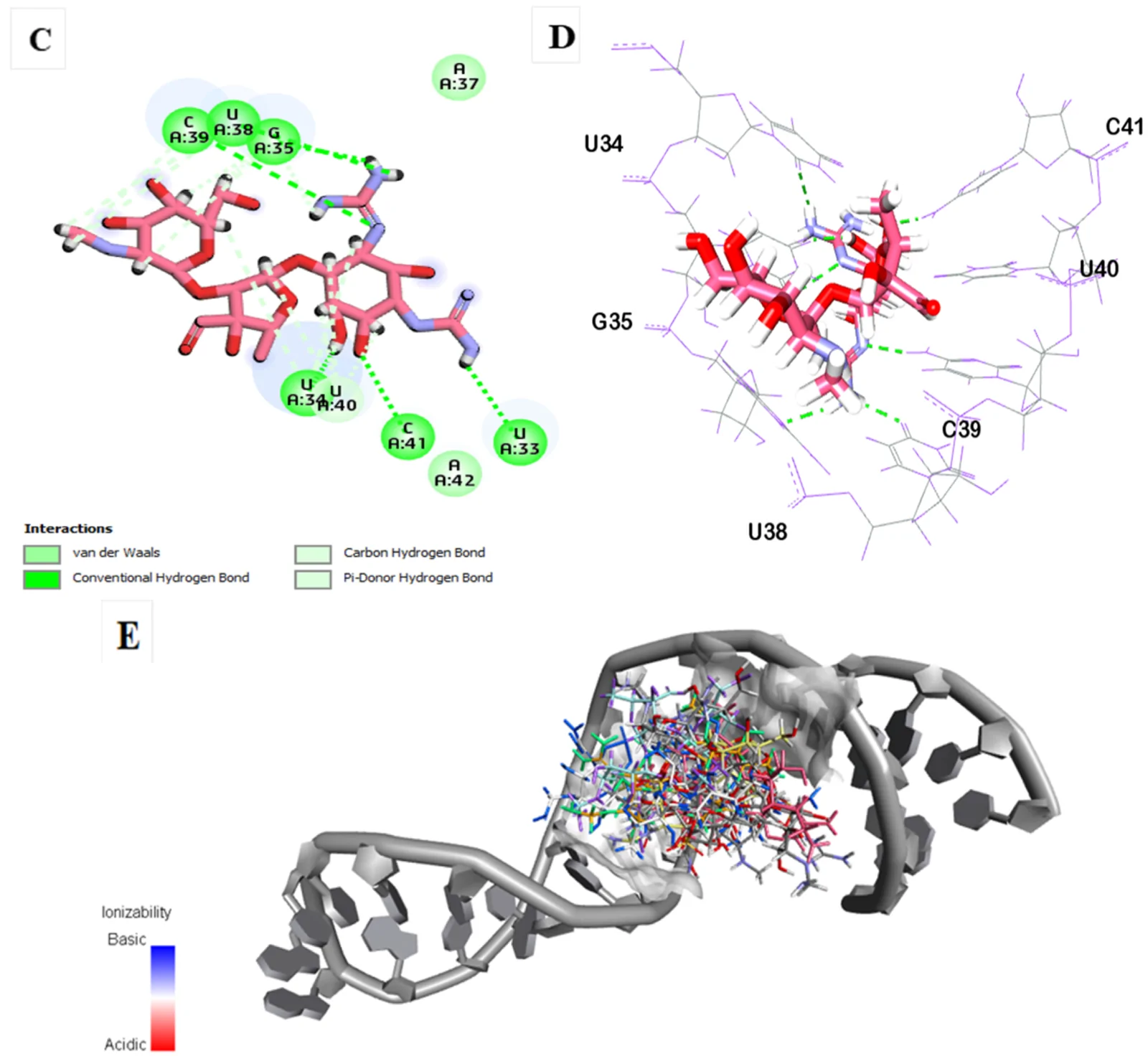

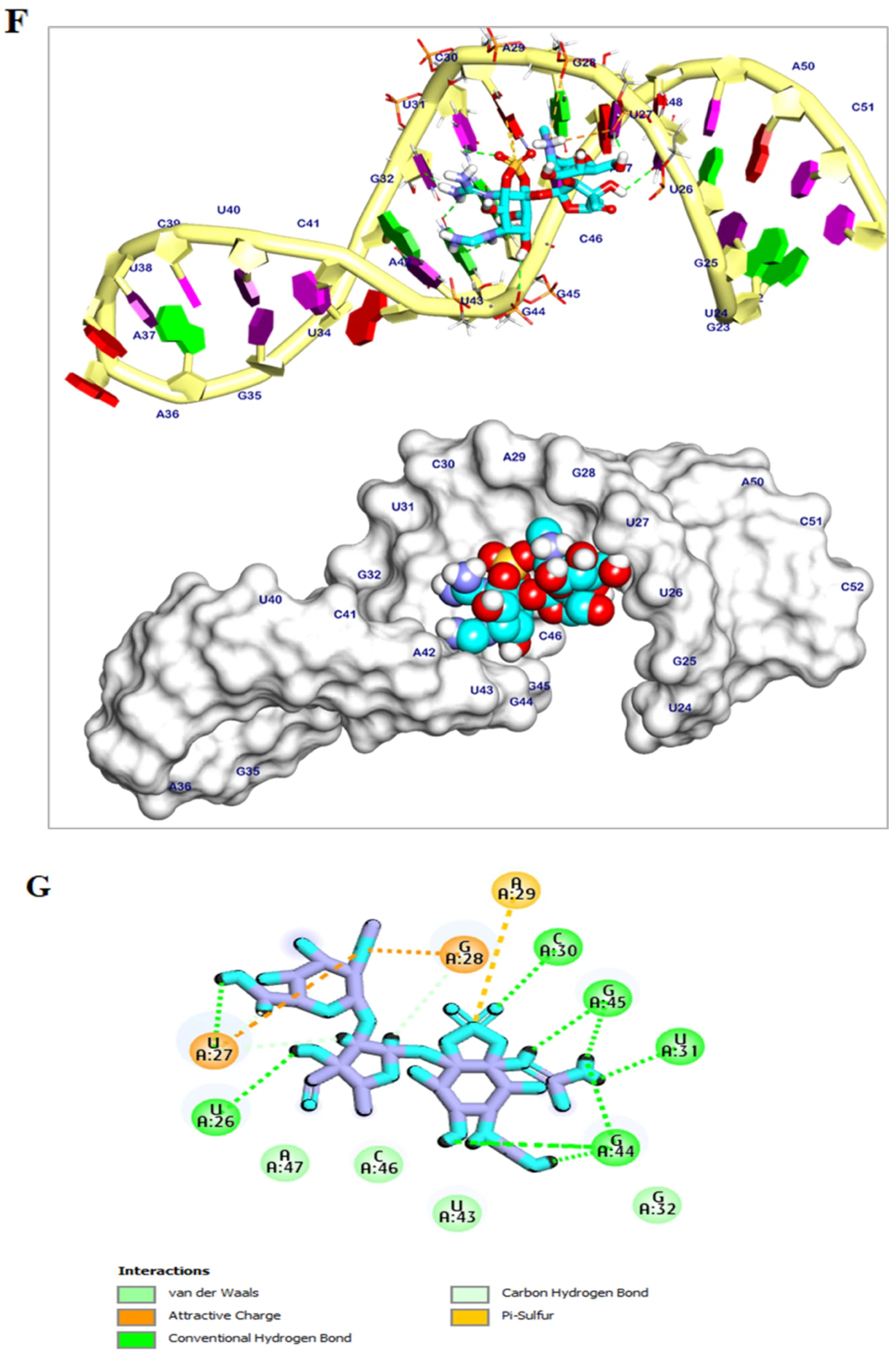

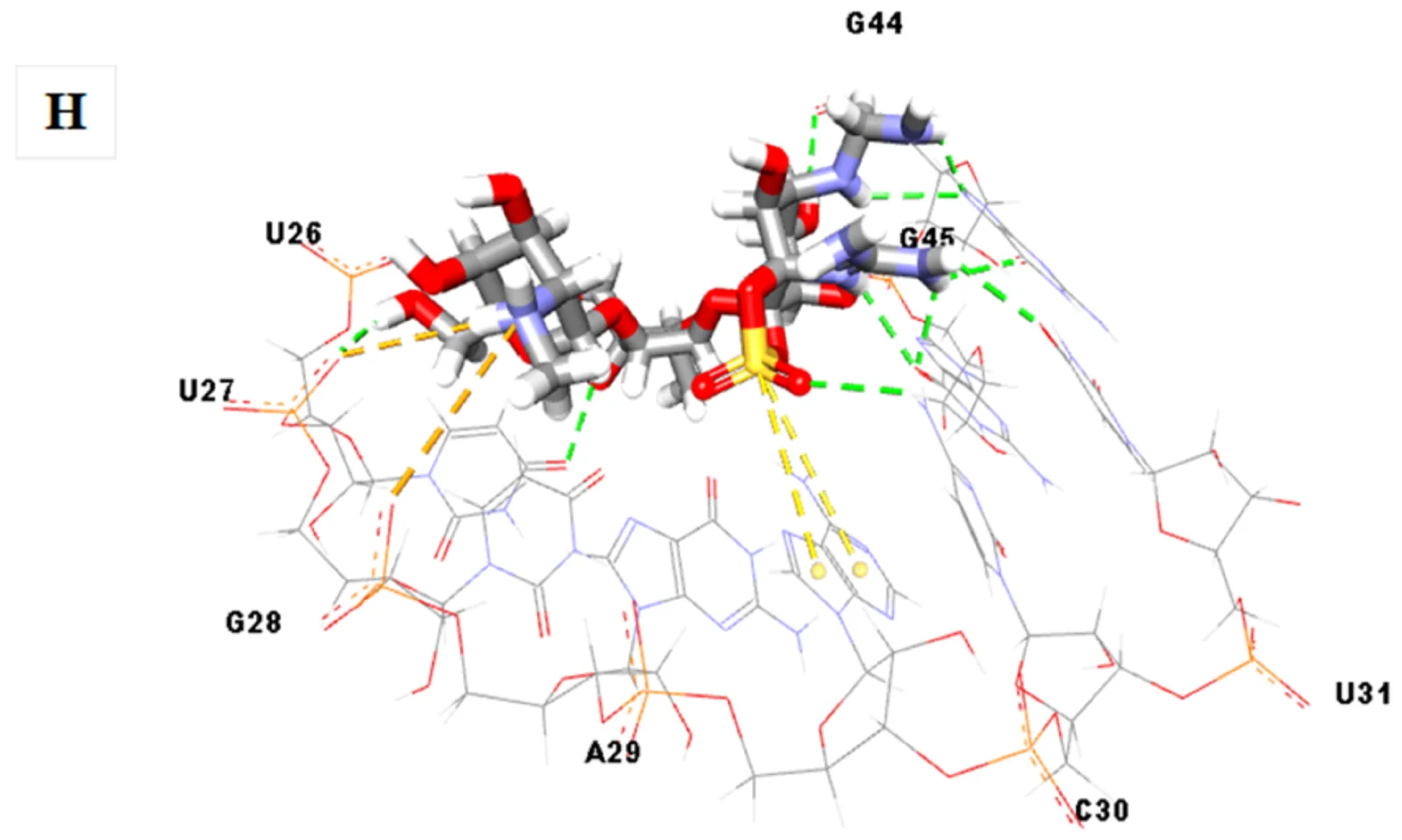
**Molecular docking of Streptomycin against precursor miR-21 target sites.** Streptomycin binding modes (shown in different colors) against the miR-21 precursor terminal loop (**A**). 3D orientation and surface mapping of Streptomycin against the miR-21 precursor terminal loop (**B**). 2D structure of Streptomycin interaction with the miR-21 precursor terminal loop (**C**); Streptomycin docked in the miR-21 precursor terminal loop (**D**), showing interacting nucleotides with 7 conventional hydrogen bonds (green color). All binding modes of Streptomycin (shown in different colors) against the miR-21 precursor at the Dicer cleavage site (**E**); 3D orientation and surface mapping against the miR-21 precursor at the Dicer cleavage site (**F**); 2D structure of Streptomycin interaction with the miR-21 precursor at the Dicer cleavage site (**G**). Streptomycin docked in the miR-21 precursor at the Dicer cleavage site (**H**), showing interacting nucleotides with 10 conventional hydrogen bonds (green color), attractive charge (orange color), and pi-sulfur interactions (yellow color).

**Figure 8:**
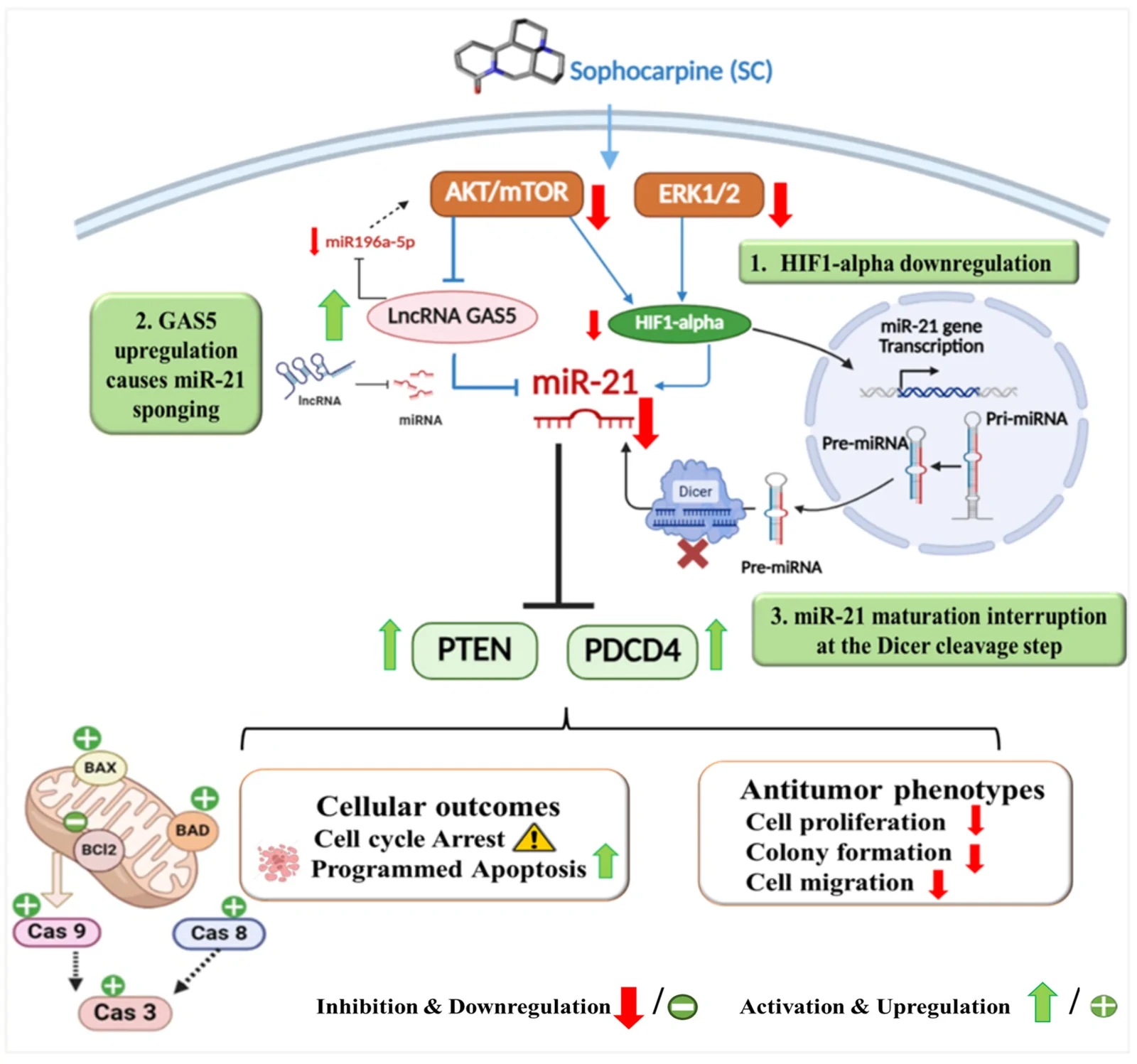
Schematic diagram for SC’s proposed molecular mechanisms in breast cancer cells.

**Table 1:** Analysis of bonds in SC and pre-miR-21 interactions.

Mode	Name	Type	Category	Distance (A_o_)	Angle DHA *	Angle HAY *	From	To
Terminal loop site (−7.22)	A:U33:H3-SC:O18	Conventional Hydrogen Bond	Hydrogen Bond	2.17948	141.851	119.847	A:U33:H3 (H-Donor)	SC:O18 (H-Acceptor)
	A:C39:H42-SC:N13	Conventional Hydrogen Bond	Hydrogen Bond	2.21849	170.145	104.3	A:C39:H42 (H-Donor)	SC:N13 (H-Acceptor)
	SC:H20-A:U40:O4	Carbon Hydrogen Bond	Hydrogen Bond	2.59471	102.266	142.054	SC:H20 (H-Donor)	A:U40:O4 (H-Acceptor)
	SC:H27-A:U40:O4	Carbon Hydrogen Bond	Hydrogen Bond	2.17397	97.726	128.516	SC:H27 (H-Donor)	A:U40:O4 (H-Acceptor)
	SC:H36-A:U38:O4	Carbon Hydrogen Bond	Hydrogen Bond	2.09959	157.78	112.506	SC:H36 (H-Donor)	A:U38:O4 (H-Acceptor)
	A:U34-:SC	Pi-Alkyl	Hydrophobic	4.6887			A:U34 (Pi-Orbitals)	SC: Alkyl
	A:G35-:SC	Pi-Alkyl	Hydrophobic	4.97521			A:G35 (Pi-Orbitals)	SC: Alkyl
	A:G35-:SC	Pi-Alkyl	Hydrophobic	4.51663			A:G35 (Pi-Orbitals)	SC: Alkyl
	A:G35-:SC	Pi-Alkyl	Hydrophobic	4.67265			A:G35 (Pi-Orbitals)	SC: Alkyl
	A:U38-:SC	Pi-Alkyl	Hydrophobic	5.47504			A:U38 (Pi-Orbitals)	SC: Alkyl
Near the stem loop (−5.8)	A:SC1:N2-A:U43:OP2	Attractive Charge	Electrostatic	5.45741			A:SC1:N2 (positive)	A:U43:OP2 (negative)
	A:A29:H61-A:SC1:O1	Conventional Hydrogen Bond	Hydrogen Bond	2.75865	113.066	154.043	A:A29:H61(H-Donor)	A:SC1:O1 (H-Acceptor)
	A:SC1:H7-A:G45:O6	Carbon Hydrogen Bond	Hydrogen Bond	2.58462	129.364	146.892	A:SC1:H7(H-Donor)	A:G45:O6 (H-Acceptor)
	A:SC1:H9-A:G45:N7	Carbon Hydrogen Bond	Hydrogen Bond	2.89546	130.314	111.992	A:SC1:H9(H-Donor)	A:G45:N7 (H-Acceptor)
	A:SC1:H15-A:G44:N7	Carbon Hydrogen Bond	Hydrogen Bond	2.43634	147.494	111.925	A:SC1:H15(H-Donor)	A:G44:N7 (H-Acceptor)
	A:SC1:N2-A:U43	Pi-Cation	Electrostatic	4.32736			A:SC1:N2 (positive)	A:U43 (Pi-Orbitals)
	A:G44-A:SC1	Pi-Alkyl	Hydrophobic	5.28109			A:G44 (Pi-Orbitals)	A:SC1 (Alkyl)
	A:G45-A:SC1	Pi-Alkyl	Hydrophobic	4.89633			A:G45 (Pi-Orbitals)	A:SC1 (Alkyl)
Near the stem loop (−5.6)	A:U43:HO2′-A:SC1:O1	Conventional Hydrogen Bond	Hydrogen Bond	2.60887	95.37	152.674	A:U43:HO2′ (H-Donor)	A:SC1:O1 (H-acceptor)
	A:SC1:HN-A:G28:O6	Conventional Hydrogen Bond	Hydrogen Bond	2.27918	145.273	123.883	A:SC1:HN (H-Donor)	A:G28:O6 (H-acceptor)
	A:U43:H2′-A:SC1:O1	Carbon Hydrogen Bond	Hydrogen Bond	2.88555	108.383	126.319	A:U43:H2′ (H-Donor)	A:SC1:O1 (H-acceptor)
	A:SC1:H9-A:G45:O6	Carbon Hydrogen Bond	Hydrogen Bond	2.63234	146.881	134.817	A:SC1:H9 (H-Donor)	A:G45:O6 (H-acceptor)
	A:SC1:H15-A:G45:O6	Carbon Hydrogen Bond	Hydrogen Bond	2.66932	135.02	133.148	A:SC1:H15(H-Donor)	A:G45:O6(H-acceptor)
	A:SC1:H13-A:U43	Pi-Sigma	Hydrophobic	2.82662			A:SC1:H13 (C-H)	A:U43 (Pi-Orbitals)
	A:G45-A:SC1:C2	Pi-Alkyl	Hydrophobic	5.36544			A:G45 (Pi-Orbitals)	A:SC1:C2 (Alkyl)

* Angle DHA: Donor-Hydrogen-Acceptor angle; * Angle HAY: Hydrogen-Acceptor-Acceptor Neighbor.

**Table 2:** Streptomycin interaction modes with pre-miR-21 target sites.

**Streptomycin (−6.47)**				
**Interacted nucleotides of pre-miR-21**	**Type**	**Distance (A** **_o_** **)**	Angle DHA *	Angle HAY *
C39	Conventional Hydrogen Bond	2.6649	123.023	95.855
C41	Conventional Hydrogen Bond	2.13193	168.575	90.371
U34	Conventional Hydrogen Bond	3.30095		
U33	Conventional Hydrogen Bond	2.66534	121.737	156.932
U34	Conventional Hydrogen Bond	2.11728	142.879	96.617
U38	Conventional Hydrogen Bond	2.30021	120.462	124.111
G35	Conventional Hydrogen Bond	2.25038	148.94	98.184
U34	Carbon Hydrogen Bond	2.42257	169.588	100.301
U40	Carbon Hydrogen Bond	2.23787	126.013	155.115
U40	Carbon Hydrogen Bond	2.45302	109.952	141.7
U40	Carbon Hydrogen Bond	2.74555	116.558	129.707
U40	Carbon Hydrogen Bond	2.60692	121.701	119.088
G35	Carbon Hydrogen Bond	2.42245	134.776	115.671
G35	Carbon Hydrogen Bond	3.02602	113.706	154.228
U34	Carbon Hydrogen Bond	2.59521	128.474	98.126
U38	Carbon Hydrogen Bond	2.79506	117.17	93.422
U38	Carbon Hydrogen Bond	2.97564	130.037	91.141
C39	Carbon Hydrogen Bond	2.43193	123.579	139.817
G35	Carbon Hydrogen Bond	2.97944	112.919	123.214
G35	Pi-Donor Hydrogen Bond	2.46254		
**Streptomycin (−7.5)**				
**Interacted nucleotides of pre-miR-21**	**Type**	**Distance (A** **_o_** **)**	**Angle DHA**	**Angle HAY**
U27	Attractive Charge	5.08421		
G28	Attractive Charge	5.01956		
C30	Conventional Hydrogen Bond	2.90734	94.343	162.096
U27	Conventional Hydrogen Bond	2.1221	145.648	131.907
U26	Conventional Hydrogen Bond	2.91274	104.742	102.341
U31	Conventional Hydrogen Bond	2.55614	129.338	141.53
G44	Conventional Hydrogen Bond	2.48892	133.358	133.47
G45	Conventional Hydrogen Bond	2.45648	123.449	118.736
G45	Conventional Hydrogen Bond	2.63802	121.623	135.064
G44	Conventional Hydrogen Bond	2.74827	148.284	134.756
G44	Conventional Hydrogen Bond	2.99896	129.263	96.123
G44	Conventional Hydrogen Bond	2.29703	132.126	114.021
G28	Carbon Hydrogen Bond	2.67806	149.265	157.946
U27	Carbon Hydrogen Bond	2.64787	142.561	114.731
U27	Carbon Hydrogen Bond	2.78094	112.713	117.96
A29	Pi-Sulfur	5.16127		
A29	Pi-Sulfur	5.47404		

* Angel DHA: Donor-Hydrogen-Acceptor angle; * Angle HAY: Hydrogen-Acceptor-Acceptor Neighbor.

**Table 3:** Gene-specific primer sequences.

Gene Name	Primer Sequence (5′ → 3′)	Accession No.	Ref.
PTEN	**F**: TGGGCCCTGTACCATCCCAAGT **R**: TGTGGCAACCACAGCCATCGT	NM_000314.4	[140]
PDCD4	**F**: ACAGGTGTATGATGTGGAGGA **R**: TTCTCAAATGCCCTTTCATCCAA	NM_001199492.2	[141]
BAX	**F**: GCGAGTGTCTCAAGCGCATC **R**: CCAGTTGAAGTTGCCGTCAGAA	NM_138761.4	[142]
BCL-2	**F**: CGGAGGCTGGGATGCCTTTGT **R**: CAAGCTCCCACCAGGGCCAAA	NM_000633.2	[143]
Bad	**F**: CCAACCTCTGGGCAGCACAGC **R**: TTTGCCGCATCTGCGTTGCTGT	NM_032989.3	[144]
LnRGAS-5	**F**: AACTTGCCTGGACCAGCTTA **R**: CAAGCCGACTCTCCATACCT	NR_152531.1	[145]
HIF1-alpha	**F**: TCACCACAGGACAGTACAGGATGC **R**: CCAGCAAAGTTAAAGCATCAGGTTCC	NM_001530.4	[146]
Caspase 3	**F**: TGTTTGTGTGCTTCTGAGCC **R**: CACGCCATGTCATCATCAAC	NM_004346.4	[147]
Caspase 9	**F**: CATTTCATGGTGGAGGTGAAG **R**: GGGAACTGCAGGTGGCTG	NM_001229.5	[147]
Caspase 8	**F**: AGAAGAGGGTCATCCTGGGAGA **R**: TCAGGACTTCCTTCAAGGCTGC	NM_001080125	[148]
GAPDH	**F:** AAGGTCGGAGTCAACGGATTTG **R:** GCCATGGGTGGAATCATATTGG	NM_001289745.3	[148]

## Data Availability

The original contributions presented in this study are included in the article/Supplementary Materials. Further inquiries can be directed to the corresponding author.

## Supplementary Materials

The following supporting information can be downloaded at https://www.mdpi.com/article/10.3390/ph19101513/s1; Table S1: Normalized Migration Index of MDA-MB-231 and T-47D cells treated with Sophocarpine (SC) for 48 h.

## Abbreviations

BAD: Bcl-2-associated agonist of cell death, Bax: Bcl-2-associated X, Apoptosis regulator, Bcl-2: B-cell lymphoma 2, VEGF: Vascular endothelial growth factor, mTORC1: Mammalian Target of Rapamycin Complex 1, TSC2: Tuberous Sclerosis Complex 2, CDK: Cyclin-dependent kinase. BSA: bovine serum albumin. Grb2: Growth factor receptor-bound protein 2. Shc: Src Homology 2 Domain-Containing; SOS: Son of Sevenless. PRAS40: Proline-Rich Akt Substrate of 40 kDa. NBT/BCIP: Nitro blue tetrazolium, BCIP: 5-bromo-4-chloro-3-indolyl phosphate.
