# Supplementary material for: Sophocarpine Inhibits Proliferation and Triggers Apoptosis in Breast Cancer Cells by Modulating the miR-21/PTEN/AKT and ERK1/2 Signaling Networks
Source: Pharmaceuticals (Basel). 2026 Sep 23;19(10):1513. doi: 10.3390/ph19101513 (PMC13708504; doi:10.3390/ph19101513)
Supplement: Supplementary file 1 [file pharmaceuticals-19-01513-s001.zip › pharmaceuticals-4481247-supplementary.pdf]

Supplementary Table S1: Normalized Migration Index of MDA-MB-231 and T-47D cells treated with Sophocarpine (SC) for 48 h.

The Normalized Migration Index was calculated by dividing Wound Closure % by Cell Viability % for each concentration at 48 hr.

$$\boldsymbol{Normalization\ \ Migration\,Index}\ (\%) = \frac{\boldsymbol{Wound\ Closure}\ \%}{\boldsymbol{Cell\,Viabilty}\ \%}\ \times \boldsymbol{100}$$

Treatment	Time	Viability %	Wound closure %	Normalized %
Untreated MDA-MB-231	**48 hr**	100	98.1 ± 0.31	98.1±0.31
SC 1.46 mM		72.5	15.2 ± 0.58	20.9±0.80***
SC 2.9 mM		50	5.8 ± 2.05	11.6±4.1***
Untreated T-47D		100	98 ± 0.02	98±0.03
SC 1.2 mM		66.9	15.4 ± 1.33	23±2.0$^{\#\#\#}$
SC 2.4 mM		50	9.1 ± 0.17	18.2±0.36$^{\#\#\#}$

*** Values are presented as mean ± SE (n=3). ****P* < 0.001 vs. untreated MDA-MB-231 cells; $^{\#\#\#}$*P* < 0.001 vs. untreated T-47D cells (determined by One-way ANOVA with Tukey's post-hoc test).**
